# LDL receptor–mediated lipoprotein uptake fuels human CD4^+^ T cell polarization toward a c-MAF/IL-10– and FOXP3-driven phenotype

**DOI:** 10.1172/jci.insight.198505

**Published:** 2026-06-08

**Authors:** Angela Markovska, Niels S. van Heusden, Dagmar Duijzer, Alejandra Bodelón, Greta Rogani, Enric Mocholi, Edwin C.A. Stigter, Can Gulersonmez, Sander Kooijman, Leonie Van der Zee, Monique T. Mulder, Jeanine E. Roeters van Lennep, Patrick C.N. Rensen, Jorg van Loosdregt, Sebastiaan J. Vastert, Noam Zelcer, Marianne Boes, Henk S. Schipper

**Affiliations:** 1 Center for Translational Immunology, University Medical Centre Utrecht, Utrecht, Netherlands.; 2 Department of Biomolecular Health Sciences, Faculty of Veterinary Medicine, Utrecht University, Utrecht, Netherlands.; 3 Center for Molecular Medicine, University Medical Center Utrecht, Regenerative Medicine Center, University Medical Center Utrecht, Utrecht, Netherlands.; 4 Department of Ciencias Biomédicas, Universidad Cardenal Herrera CEU, Valencia, Spain.; 5 Center for Molecular Medicine, University Medical Center Utrecht, Oncode Institute, Utrecht, Netherlands.; 6 Department of Medicine, Division of Endocrinology, and Einthoven Laboratory for Experimental Vascular Medicine, Leiden University Medical Center, Leiden, Netherlands.; 7 Department of Internal Medicine Laboratory, Erasmus MC Cardiovascular Institute, Erasmus MC, University Medical Center Rotterdam, Rotterdam, Netherlands.; 8 Department of Pediatric Rheumatology and Immunology, University Medical Center Utrecht, Utrecht, Netherlands.; 9 Department of Medical Biochemistry, Amsterdam UMC — location AMC, University of Amsterdam, Amsterdam, Netherlands.; 10 Amsterdam Gastroenterology, Endocrinology, and Metabolism Institute, Amsterdam UMC, Amsterdam, Netherlands.; 11 Amsterdam Cardiovascular Sciences Institute, Amsterdam UMC, Amsterdam, Netherlands.; 12 Pediatrics Department, University Medical Center, Utrecht, Netherlands.; 13 Department of Pediatric Cardiology, Sophia Children’s Hospital, Erasmus Medical Center, Rotterdam, Netherlands.

**Keywords:** Cell biology, Immunology, Lipoproteins, T cells

## Abstract

Human CD4^+^ T cells utilize nutrients, including lipids, to support their activation and polarization. Considering the pivotal role of lipoproteins in lipid transport, we reasoned that lipoprotein uptake and processing could effect CD4^+^ T cell function. Here, we demonstrate that activation of human CD4^+^ T cells induced expression of LDL receptor (LDLR) to facilitate LDLR-mediated endocytosis of LDL. Degradation of surface LDLR on CD4^+^ T cells with PCSK9 hampered activation and proliferation of the cells. Lipoprotein deprivation or blocking of lysosomal cholesterol egress impaired activation of mechanistic target of rapamycin complex 1 (mTORC1), affecting CD4^+^ T cell activation and proliferation. Furthermore, lipoprotein deprivation of cultured primary CD4^+^ T cells lead to reduced expression of c-MAF and FOXP3, key transcription factors for IL-10, accompanied by reduced IL-10 secretion. The pivotal role of LDLR-mediated lipoprotein uptake for mTORC1 activity, c-MAF and FOXP3 expression, and IL-10 secretion was confirmed using LDLR-dysfunctional CD4^+^ T cells from patients with homozygous familial hypercholesterolemia. Our study offers valuable insights into the lipoprotein metabolism of human CD4^+^ T cells and their reliance on the LDLR pathway for activation and polarization, a feature that may be leveraged to modulate CD4^+^ T cell function.

## Abstract 2

CD4+ T cells require lipoprotein uptake via LDL receptors to support mTORC1 signaling, activation, proliferation, and production of the anti-inflammatory cytokine IL-10.

## Introduction

CD4^+^ T cells use diverse nutrients, including glucose, amino acids, and lipids such as fatty acids and cholesterol, to meet increases in energy demand upon activation (1, 2). Recent insights from immunometabolism research reveal that nutrients are not merely an energy source, they also regulate key signaling pathways shaping T cell fate and function (3–10). Glucose is rapidly metabolized through glycolysis, for example, to support the generation of effector T cells and translation and secretion of cytokines such as IFN-γ (11). Additionally, fatty acids undergo β oxidation and oxidative phosphorylation to generate substantial amounts of ATP, which has been implicated in skewing T cell responses toward a regulatory phenotype (12). In addition to nutrient types, the mere availability of nutrients also influences CD4^+^ T cell responses. In the tumor microenvironment, competition for glucose and lipids depletes these resources, impairing the function of tumor-infiltrating CD8^+^ T cells (13). As an opposing example, elevated levels of oxidized lipids in atherosclerotic plaques can exacerbate the inflammatory phenotype of CD4^+^ T cells (14). Thus, nutrient types and their availability are clearly important determinants for T cell function.

Cells obtain cholesterol from two main sources, endogenous synthesis and exogenous uptake (15). Endogenously, cells synthesize cholesterol de novo starting from acetyl-CoA through a multistep enzymatic pathway, which is tightly regulated to maintain cellular cholesterol homeostasis (16). Exogenously, cells acquire lipids by internalizing circulating lipoproteins. Lipoproteins are multimolecular particles that function as carriers of hydrophobic lipid molecules through aqueous fluids in the human body. Among the various types of lipoproteins (i.e., chylomicrons, VLDLs, LDLs, and HDLs), LDL particles carry the highest cholesterol content and can vary in concentration depending on the individual, the tissue, and the physiological or pathological state (17, 18). Beyond their classical role in lipid transport, LDL particles can modulate the function of various cell types, including endothelial cells, hepatocytes, and immune cells such as macrophages and CD8^+^ T cells (19–21). For example, LDLR-mediated lipoprotein uptake has been shown to be critical for mouse CD8^+^ T cell effector functions, partly through its interactions with the mechanistic target of rapamycin complex 1 (mTORC1) (22). As a central regulator of cell metabolism and growth, mTORC1 integrates nutrient and energy signals to coordinate cellular functions. In CD4^+^ T cells, mTORC1 is critical for cellular differentiation and function (23–25). Recent research has revealed that mTORC1 activation requires cholesterol derived from lysosomes, which serves as a key metabolic signal to promote the complex’s assembly and function (26–28). As the main source of lysosome-derived cholesterol needed for mTORC1 activation comes from LDL receptor–mediated (LDLR-mediated) lipoprotein uptake, we reasoned that lipoproteins have a role in the immune functions of CD4^+^ T cells.

Here, we describe how LDLR-mediated lipoprotein uptake shaped human CD4^+^ T cell activation and polarization by using various experimental models, including proprotein convertase subtilisin/kexin type 9–mediated (PCSK9-mediated) LDLR degradation, lipoprotein deprivation, inhibition of lipoprotein processing, and LDLR-deficient CD4^+^ T cells from patients with homozygous familial hypercholesterolemia (hoFH). We demonstrate that LDLR-mediated LDL uptake by CD4^+^ T cells was critical for sustaining mTORC1 signaling and the expression of the transcription factors c-MAF and FOXP3, which are key regulators of IL-10 production. Accordingly, CD4^+^ T cells with impaired LDL uptake or LDLR deficiency exhibited reduced IL-10 secretion. Our data reveal a critical role of LDLR-mediated lipoprotein uptake in shaping function and signaling pathways in human CD4^+^ T cells.

## Results

### Activated CD4^+^ T cells upregulate LDLR expression and lipoprotein metabolism.

**Figure 1:**
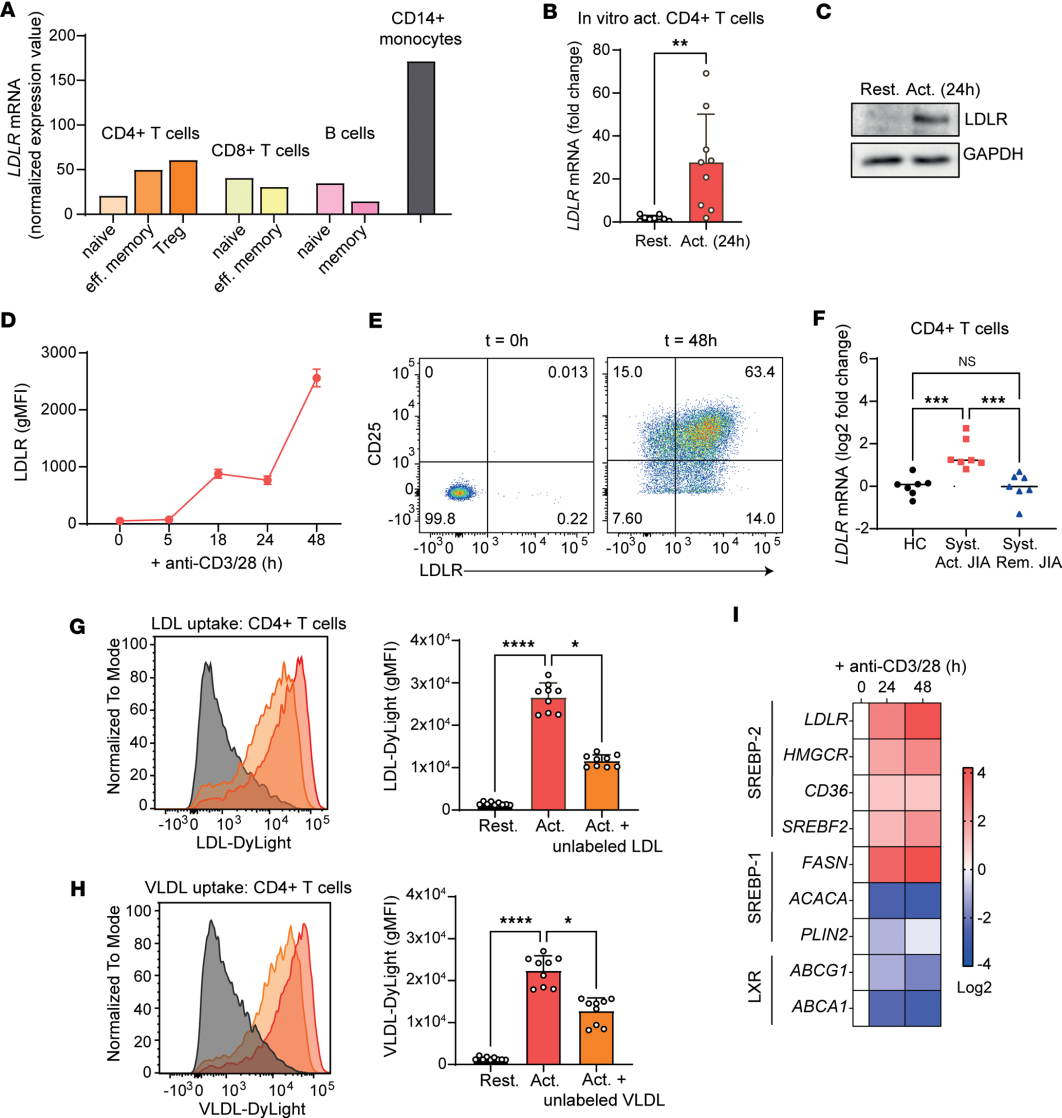
Activated CD4^+^ T cells upregulate LDLR expression and lipoprotein metabolism. (**A**) Normalized expression of *LDLR* mRNA values among immune cell types obtained from the Human Cell Atlas (https://data.humancellatlas.org/). Effector memory T cells were defined as CD127^+^CD25^–^CD62L^–^CD45RA^–^, naive T cells as CD127^+^CD25^–^CD62L^+^CD45RA^+^, and Tregs as CD4^+^CD25^+^CD127^–^. (**B**) *LDLR* mRNA normalized to *RPL13A* and shown relative to resting cells in CD4^+^ T cells from healthy donors activated for 24 hours with anti-CD3/CD28. Two-tailed unpaired *t* test (**P < 0.01; *n* = 9). (**C**).Western blot of LDLR and GAPDH (loading control) in CD4^+^ T cells activated for 24 hours with anti-CD3/CD28. (**D**) Geometric mean fluorescence intensity (gMFI) of cell-surface LDLR on CD4^+^ T cells. (**E**) Representative flow cytometry plot of CD25 and LDLR staining of CD4^+^ T cells at resting (*t* = 0) or after 48 hours of activation (*t* = 48). (**F**) Log_2_ fold change of *LDLR* in ex vivo blood-derived CD4^+^ T cells from patients with systemic juvenile idiopathic arthritis (JIA) in active (*n* = 7) or remission state (*n* = 7) relative to the CD4^+^ T cells from the individuals acting as healthy controls (HC) (*n* = 7). One-way ANOVA with Tukey’s multiple comparisons test (***P < 0.001). (**G** and **H**) Representative histograms of CD4^+^ T cells loaded with LDL/VLDL-DyLight and quantification of the gMFI of the signal. Cells were activated (anti-CD3/CD28) and loaded with LDL/VLDL-DyLight, with or without unlabeled lipoproteins, for 16 hours in lipoprotein-deprived medium. Kruskal-Wallis test with Dunn’s multiple comparisons test (*P < 0.05, ****P < 0.0001; *n* = 9). (**I**) qRT-PCR analysis of genes involved in lipoprotein uptake and metabolism in CD4^+^ T cells from healthy donors at the indicated times after stimulation with anti-CD3/CD28. Values are normalized to *RPL13A* endogenous control and shown relative to resting cells (*n* = 3).

Upon activation, CD4^+^ T cells upregulate several metabolic pathways to fulfil their nutrient requirements (1, 2, 9). To study LDLR expression in human CD4^+^ T cells, we first analyzed publicly available gene expression datasets, using monocytes as a positive control for their established LDLR expression and functional relevance (29, 30). We found that human CD4^+^ T cells express *LDLR* levels comparable to those of CD8^+^ T cells and B cells. (Figure 1A). The *LDLR* mRNA expression was particularly high in effector memory and regulatory CD4^+^ T cells.

As previous studies showed a functional effect of LDL uptake on CD8^+^ T cells (22) and B cells (31–33), we next investigated LDLR expression upon in vitro activation (CD3/CD28 Dynabeads, 24 hours) of CD4^+^ T cells from healthy donors. In vitro activated cells showed higher *LDLR* mRNA compared with resting cells (Figure 1B). Moreover, activation of CD4^+^ T cells led to a rise in total LDLR protein (Figure 1C), as shown by immunoblotting, and an increased LDLR surface abundance (Figure 1D), as determined by flow cytometry. Costaining the CD4^+^ T cells with the activation marker CD25 revealed that the majority of CD25^+^ cells also exhibited high LDLR expression 48 hours after in vitro activation (Figure 1E). To assess *LDLR* expression in activated CD4^+^ T cells in vivo, we analyzed samples from patients with systemic juvenile idiopathic arthritis (JIA) during active disease or remission. CD4^+^ T cells from patients with active JIA displayed increased activation-associated gene expression and higher abundance of activation markers compared with individuals acting as healthy controls and patients in remission (Supplemental Figure 1, A and B; supplemental material available online with this article; https://doi.org/10.1172/jci.insight.198505DS1). *LDLR* mRNA expression was elevated in CD4^+^ T cells during active disease and normalized in remission (Figure 1F). Consistently, CD4^+^ T cells isolated from synovial fluid of children with active oligoarticular JIA showed enrichment of T cell activation-related transcriptional programs and increased *LDLR* mRNA expression compared with CD4^+^ T cells from healthy control blood (Supplemental Figure 1, C and D). Together, these data demonstrate increased *LDLR* expression in activated CD4^+^ T cells in vivo.

Next, we studied whether the increased LDLR expression after activation would result in increased LDL uptake. For this, we cultured CD4^+^ T cells with DyLight-labeled LDL or VLDL and measured the resulting intracellular fluorescent signal using flow cytometry. In vitro activated CD4^+^ T cells had an increase in LDL and VLDL uptake compared with resting cells (Figure 1, G and H). As a control, uptake of the labeled lipoproteins was reduced when excess unlabeled lipoproteins were added to the culture medium.

The key transcription factors regulating intracellular lipid metabolism genes are SREBP-1, SREBP-2, and liver X receptor α and β (LXRs). SREBP-1 primarily regulates genes involved in fatty acid and triglyceride synthesis, SREBP-2 controls cholesterol biosynthesis and uptake, and LXRs regulate genes involved in cellular cholesterol efflux (34). To better understand the dynamic regulation of lipid metabolism, we measured mRNA levels of genes involved in lipid metabolism in in vitro activated CD4^+^ T cells. Our analysis suggests that genes primarily driven by SREBP-2 are upregulated upon activation (Figure 1I).

Taken together, activated human CD4^+^ T cells upregulate LDLR expression and lipoprotein uptake and metabolism, which coincides with increased expression of SREBP-2–driven genes.

### SREBP-2 drives endocytosis and lysosomal processing of lipoproteins by CD4^+^ T cells.

**Figure 2:**
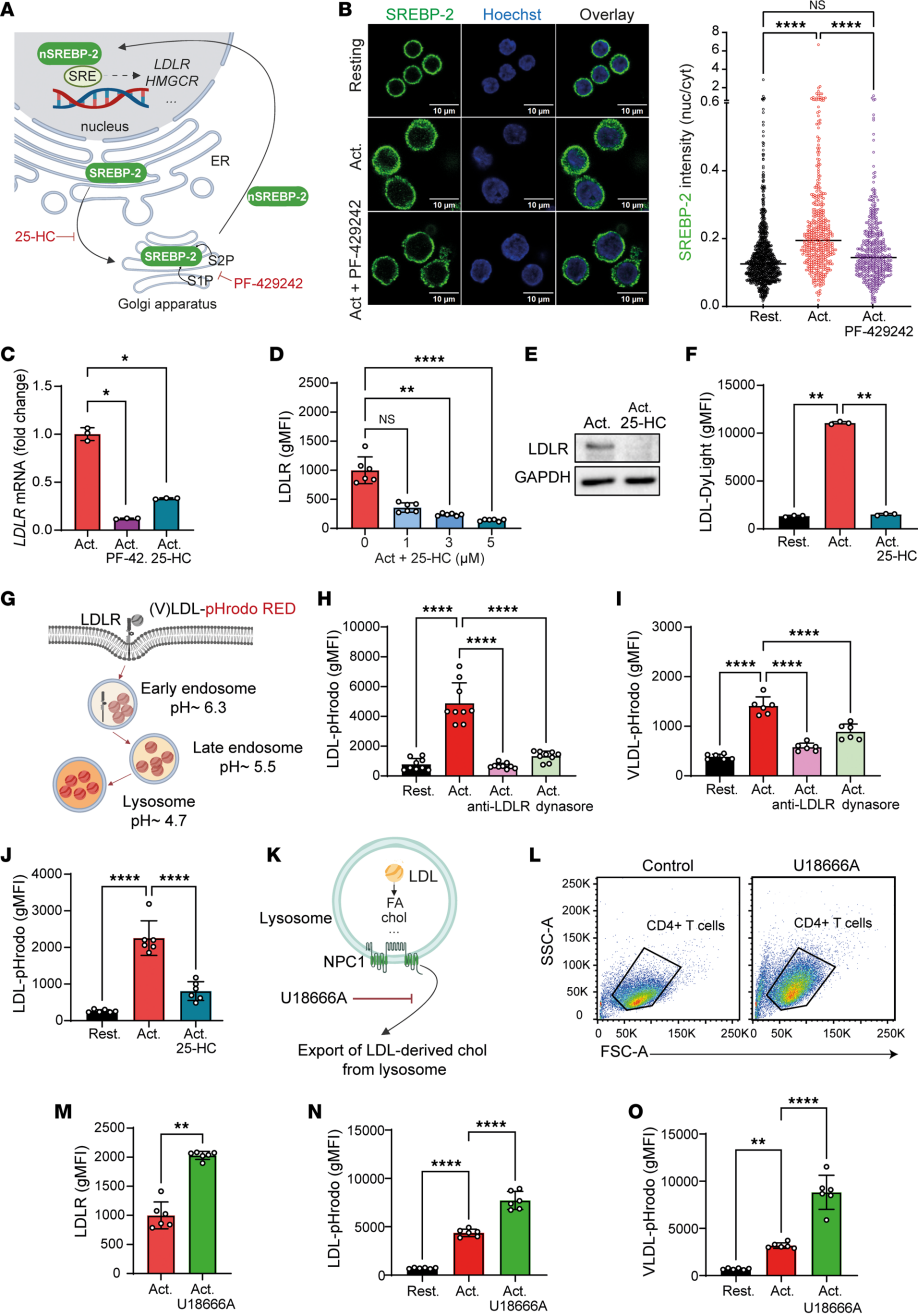
SREBP-2 drives endocytosis and lysosomal processing of lipoproteins by activated CD4^+^ T cells. (**A**) Schematic of SREBP-2 pathway. (**B**) Representative confocal images of SREBP-2 and Hoechst staining in resting or activated CD4^+^ T cells (anti-CD3/CD28, 24 hours). Where indicated, cells were treated with PF-429242 (10 μM). Quantification of the SREBP-2 nuclear/cytoplasmic intensity (*n* = 3; 5 images/condition). Kruskal-Wallis test with Dunn’s multiple comparisons test (****P < 0.0001). Scale bar: 10 �m. (**C**) *LDLR* mRNA in CD4^+^ T cells activated for 24 hours with and without 25-HC (5 μM) or PF-429242 (10 μM). Kruskal-Wallis with Dunn’s test (*P < 0.05; *n* = 3). (**D**) Cell surface LDLR on CD4^+^ T cells activated for 24 hours. Kruskal-Wallis with Dunn’s multiple comparisons test (**P < 0.01, ****P < 0.0001; *n* = 6). (**E**) LDLR and GAPDH levels measured by Western blot in activated CD4^+^ T cells (anti-CD3/CD28, 24 hours). (**F**) Cells were activated and loaded with LDL-DyLight for 16 hours. Where indicated, cells were treated with 25-HC. Kruskal-Wallis with Dunn’s multiple comparisons test (**P < 0.01; *n* = 3). (**G**) Schematic of the lipoprotein-pHrodo uptake. Uptake of (**H**) LDL-pHrodo and (**I**) VLDL-pHrodo measured by flow cytometry, where CD4^+^ T cells were activated for 24 hours, followed by a 2-hour culture in lipoprotein-deprived medium and 2-hour incubation with lipoprotein-pHrodo. One-way ANOVA with Dunnett’s multiple comparisons test (****P < 0.0001); *n* = 9 (**H**) and *n* = 6 (**I**). (**J**) Cells were activated, where indicated treated with 25-HC for 24 hours, followed by 2-hour LDL-pHrodo loading. One-way ANOVA with Tukey’s multiple comparisons test (****P < 0.0001; *n* = 6). (**K**) Schematic of NPC1 and U18666A. (**L**) Forward-scatter area (FSC-A)/side-scatter area (SSC-A) plots of CD4^+^ T cells activated for 24 hours with or without U18666A (2 μg/mL). (**M**) Cell surface LDLR expression on CD4^+^ T cells measured by flow cytometry. Mann-Whitney *U* test (**P < 0.01; *n* = 6). (**N** and **O**) Uptake of LDL-pHrodo and VLDL-pHrodo by CD4^+^ T cells. Ordinary 1-way ANOVA with Dunnett’s multiple comparisons test (**P < 0.01, ****P < 0.0001; *n* = 6).

SREBP-2 is the main transcription factor regulating the expression of *LDLR*, as shown in cells such as hepatocytes and macrophages (35–37). Upon decreased cholesterol levels within the endoplasmic reticulum (ER), SREBP-2 protein translocates to the Golgi apparatus, where it undergoes sequential proteolytic cleavage by site-1 and -2 proteases to release its active transcriptional domain (16, 38). Subsequently, active SREBP-2 migrates to the nucleus, binding to sterol-responsive elements on the promoters of target genes, including *LDLR*. 25-hydroxycholesterol (25-HC) can inhibit anterograde trafficking of the SREBP-2/SCAP complex from the ER, while PF-429242 inhibits the cleavage of SREBP-2 by site-1 protease into its active form (Figure 2A). To investigate whether activation of CD4^+^ T cells affects SREBP-2 activity, we stained the cells with an antibody targeting the N-terminal region of SREBP-2, present in both its inactive precursor (ER/cytoplasm) and active (nuclear) forms, and with Hoechst nuclear dye. We visualized the cells with confocal microscopy and quantified the nuclear/cytoplasmic intensity of the SREBP-2 staining. The nuclear/cytoplasmic SREBP-2 signal was higher in activated cells compared with that in resting cells, suggesting that CD4^+^ T cell activation is associated with increased active (nuclear) SREBP-2 localization (Figure 2B). Treatment of activated CD4^+^ T cells with SREBP-2 inhibitor PF-429242 decreased the nuclear/cytoplasmic SREBP-2 signal to resting levels (Figure 2B). Next, we measured LDLR levels in activated cells with restricted SREBP-2 activation owing to 25-HC and PF-429442 treatment. *LDLR* mRNA (Figure 2C), cell surface occupancy (Figure 2D), and total protein level (Figure 2E) were decreased when SREBP-2 activation was attenuated in CD4^+^ T cells. Moreover, inhibiting activation of SREBP-2 with 25-HC decreased LDL uptake by cells, as measured by assessing LDL-DyLight uptake with flow cytometry (Figure 2F).

To further study the endolysosomal processing of lipoproteins, we conjugated LDL and VLDL particles with pHrodo dye, which exhibits increased fluorescence as the pH decreases. We activated CD4^+^ T cells for 24 hours, followed by a 2-hour period of lipoprotein deprivation, and subsequently cocultured the cells with the fluorescent pHrodo-labeled lipoproteins for 2 hours. Flow cytometry was then used to measure lipoprotein uptake, in which we included two negative controls: cells pretreated with either a blocking anti-LDLR antibody or dynasore (inhibitor of dynamin, involved in endocytosis) (39). Since the endolysosomal compartment undergoes gradual acidification, internalized lipoproteins were expected to show increased fluorescence (Figure 2G). Activated CD4^+^ T cells had higher V/LDL-pHrodo uptake compared with resting cells (Figure 2, H and I). Preincubation with a blocking antibody against LDLR or treatment of cells with dynasore reduced V/LDL-pHrodo uptake to resting levels (Figure 2, H, and I). Next, we asked if SREBP-2 activation drives the observed LDLR-mediated lipoprotein uptake in activated cells. To address this question, we pretreated the cells with 25-HC, followed by the LDL-pHrodo uptake assay. Activated cells pretreated with 25-HC showed reduced LDL-pHrodo uptake compared with untreated activated cells (Figure 2J). Thus, our data demonstrate that SREBP-2 drives the LDLR-mediated endocytosis of lipoproteins by activated CD4^+^ T cells.

Within the lysosome, LDL particles undergo enzymatic degradation, releasing free cholesterol and fatty acids, among other molecules. The cellular use of cholesterol requires its egress from the lysosome, which is facilitated by the Niemann-Pick type C1 (NPC1) protein. Accordingly, NPC1 inhibitors, such as U18666A, impair lysosomal cholesterol egress and lead to lysosomal cholesterol accumulation (40) (Figure 2K). To address this possibility in CD4^+^ T cells, we activated the cells in presence of the NPC1 inhibitor U18666A, followed by measurement of endolysosomal LDL-pHrodo by flow cytometry. CD4^+^ T cells activated in presence of U18666A had increased side scatter area, a reflection of increased cellular granularity, as measured by flow cytometry (Figure 2L). Furthermore, CD4^+^ T cells activated in the presence of U18666A exhibited increased LDLR expression on their cell surface, suggesting enhanced SREBP-2 activity resulting from decreased extralysosomal cellular cholesterol (Figure 2M). Finally, NPC1 inhibition with U18666A led to increased endolysosomal presence of V/LDL-pHrodo compared with the untreated condition in activated CD4^+^ T cells (Figure 2, N and O).

Taken together, activated CD4^+^ T cells have increased SREBP-2 transcriptional activity, which promotes LDLR-mediated endocytosis of lipoproteins. These lipoproteins are subsequently processed, and NPC1 is involved in the egress of LDL-derived cholesterol from the lysosomal compartment.

### LDLR-mediated lipoprotein uptake fuels CD4^+^ T cell activation and proliferation.

To determine whether LDL uptake and processing affect CD4^+^ T cell activation and proliferation in vitro, we activated CD4^+^ T cells under 4 conditions: (a) control media containing all lipoproteins; (b) media with U18666A to inhibit NPC1, blocking lysosomal cholesterol egress and cellular use of lipoprotein-derived cholesterol; (c) lipoprotein-deprived media; and (d) lipoprotein-deprived media supplemented with LDL.

**Figure 3:**
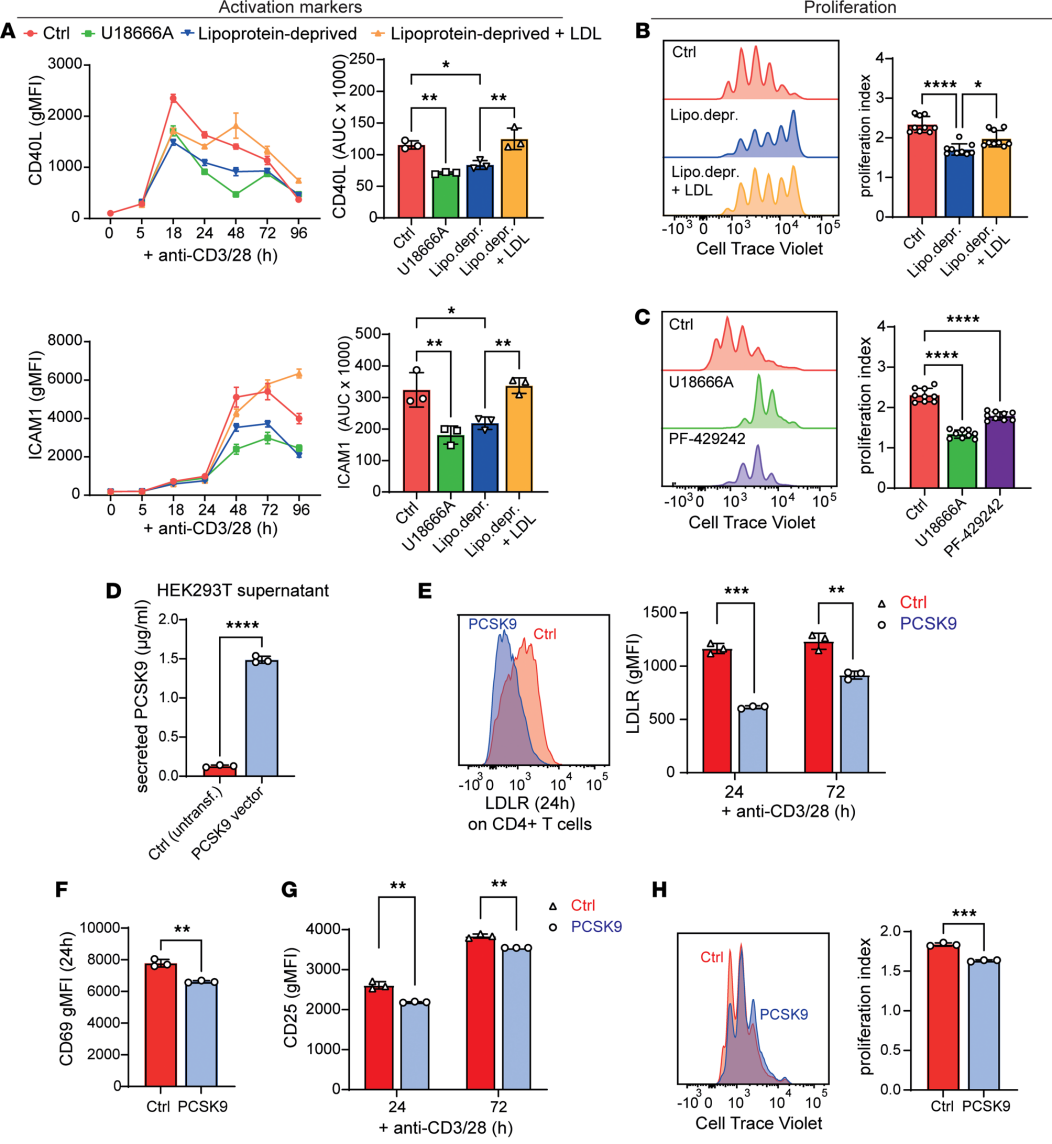
LDLR-mediated lipoprotein uptake by CD4^+^ T cell fuels their activation and proliferation. CD4^+^ T cells were activated with anti-CD3/CD28 Dynabeads and cultured in control (ctrl) medium, lipoprotein-deprived medium, or lipoprotein-deprived medium supplemented with LDL (10 μg/mL). Where indicated, cells were treated with U18666A (2 μg/mL) or PF-420242 (10 μM). (**A**) Cell surface expression of CD40L and ICAM1 measured with flow cytometry. The statistical analysis was done on the area under the curve (AUC). One-way ANOVA with Šídák’s multiple comparisons test, (*P < 0.05, **P < 0.01; *n* = 3). (**B** and **C**) Representative flow cytometry histograms of the CellTrace Violet dilution. For quantification, we show the proliferation index calculated by the proliferation tool (FlowJo). (**B**) Kruskal-Wallis with Dunn’s multiple comparisons test (*P < 0.05, ****P < 0.0001; *n* = 9). (**C**) One-way ANOVA with Dunnett’s multiple comparisons test (***P < 0.001; *n* = 9). (**D**) PCSK9 levels measured with ELISA in the supernatant from HEK293T cells added to the CD4^+^ T cells (nontransfected cell line and cell line overexpressing PCSK9). Cells were cultured in T cell medium for 24 hours before starting to add the supernatant to the CD4^+^ T cells. (**E**) LDLR cell surface levels measured on CD4^+^ T cells with flow cytometry. Cells were activated with anti-CD3/CD28 Dynabeads for 24 or 48 hours and cultured in supernatant from HEK293T cells (nontransfected cell line and cell line overexpressing PCSK9). (**F**) CD69 and (**G**) CD25 cell surface levels measured on the CD4^+^ T cells with flow cytometry. (**H**) Representative flow cytometry histograms of CellTrace Violet dilution. Cells were activated for 4 days with anti-CD3/CD28. For quantification, we show the proliferation index calculated by the proliferation tool (FlowJo). (**D**–**H**) Unpaired *t* tests (**P < 0.01, ***P < 0.001, ****P < 0.0001; *n* = 3).

We quantified the surface abundance of activation markers (CD40L, ICAM-1, OX40, HLA-DR and CD25) using flow cytometry over a 96-hour CD4^+^ T cell activation period. CD4^+^ T cells activated in the lipoprotein-deprived condition exhibited reduced expression of all activation markers tested compared with the control condition (Figure 3A and Supplemental Figure 2A). Reintroducing LDL to the lipoprotein-deprived medium increased overall activation marker expression (Supplemental Figure 2A), with an enhancement of CD40L and ICAM-1 (Figure 3A). Similar to the lipoprotein-deprived condition, U18666A treatment reduced surface abundance of the activation markers (Figure 3A and Supplemental Figure 2A). To measure cellular proliferation, we labeled the CD4^+^ T cells with CellTrace Violet and measured the fluorescence signal on flow cytometry. CD4^+^ T cells activated under lipoprotein-deprived conditions had impaired proliferation, and supplementation with LDL largely restored proliferation (Figure 3B). Inhibition of SREBP-2 activation with PF-429242 and inhibition of NPC1 with U18666A both impaired CD4^+^ T cells proliferation (Figure 3C). Given that SREBP-2 regulates both *LDLR* expression and cholesterol biosynthesis, inhibition of cholesterol synthesis with simvastatin increased LDLR expression on CD4^+^ T cells, and lipoprotein deprivation induced *HMGCR* upregulation; however, neither response was sufficient to restore T cell activation or proliferation (Supplemental Figure 2, B–E). Thus, perturbation of cholesterol metabolism compromises CD4^+^ T cell activation and proliferation.

To test whether the observed effects of lipoprotein availability were dependent on LDLR-mediated uptake, we employed an additional experimental model in which LDLR was selectively downregulated using PCSK9. Treatment with PCSK9-enriched supernatant (Figure 3D) induced degradation of surface LDLR on CD4^+^ T cells without affecting cell viability, resulting in significantly reduced LDLR expression at both 24 and 72 hours following activation compared with control-treated cells (Figure 3E and Supplemental Figure 2F). Consistent with the phenotypes observed under lipoprotein-deprived and NPC1-inhibited conditions, PCSK9-treated CD4^+^ T cells exhibited reduced expression of activation markers, including CD25 and CD69 (Figure 3, F and G), as well as impaired proliferative capacity (Figure 3H). Together, these findings provide complementary evidence that efficient LDL uptake via LDLR is required for optimal CD4^+^ T cell activation and proliferation.

### Lipoprotein metabolism by CD4^+^ T cells is required for c-MAF/IL-10– and FOXP3-driven phenotype.

**Figure 4:**
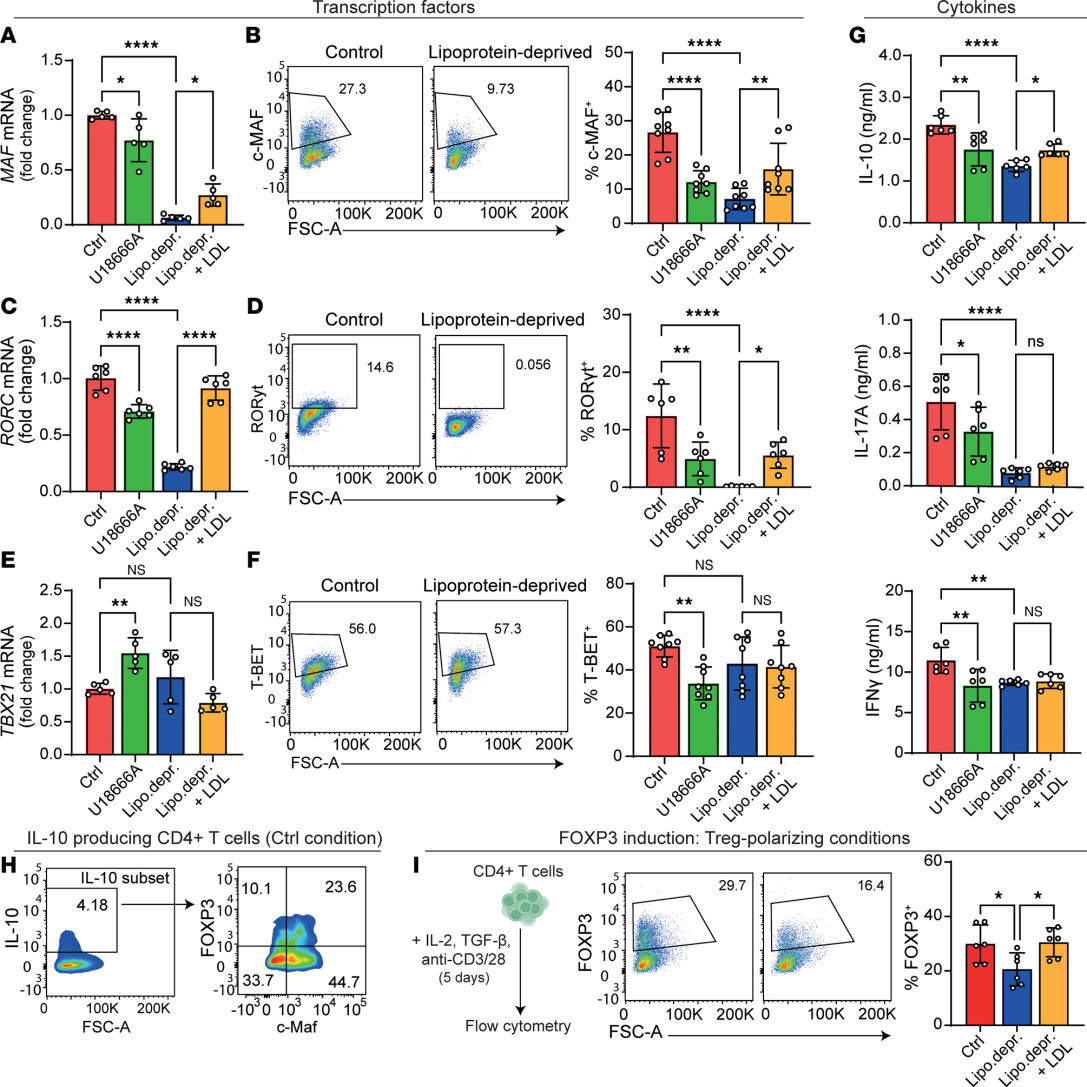
Lipoproteins fuel CD4^+^ T cell polarization. CD4^+^ T cells were activated with anti-CD3/CD28 Dynabeads and cultured in control (ctrl) medium, lipoprotein-deprived medium, or lipoprotein-deprived medium supplemented with LDL (10 μg/mL). Where indicated, cells were treated with U18666A (2 μg/mL) or PF-420242 (10 μM). (**A**–**F**) CD4^+^ T cells activated with anti-CD3/CD28 Dynabeads for 48 hours. (**A**, **C**, and **E**) *cMAF*, *RORC*, and *TBX21* mRNA levels measured in CD4^+^ T cells with qPCR and normalized to RPL13A levels relative to the ctrl condition. One-way ANOVA with Šídák’s multiple comparisons test (*P < 0.05, **P < 0.01 ****P < 0.0001; *n* = 5) in **A** and **E** and *n* = 6 in **C**. (**B**, **D**, and **F**) Intracellular protein expression of c-MAF, RORγt, and T-BET measured with flow cytometry. One-way ANOVA with Šídák’s multiple comparisons test (*P < 0.05, **P < 0.01, ****P < 0.0001); *n* = 8 in **B** and **F** and *n* = 6 in **D**. (**G**) IFN-γ, IL-17A, and IL-10 concentrations in supernatant of CD4^+^ T cells activated for 24 hours measured by ELISA. One-way ANOVA with Šídák’s multiple comparisons test (*P < 0.05, **P < 0.01, ****P < 0.0001; *n* = 6). (**H**) Representative flow cytometric analysis from CD4^+^ T cells activated for 24 hours with anti-CD3/CD28 and stained intracellularly for IL-10, c-MAF, and FOXP3. (**I**) CD4^+^ T cells were cultured in Treg-polarizing conditions with IL-2 and TGF-β for 5 days, after which FOXP3 expression was measured by flow cytometry. One-way ANOVA with Dunnett’s multiple comparisons test (*P < 0.05; *n* = 6).

Next, we investigated how lipoprotein uptake affects CD4^+^ T cell function by measuring the expression of transcription factors for key cytokine genes (41). Previous research has shown that cholesterol flux within T cells regulates the expression of c-MAF (42), a transcription factor that directly regulates IL-10 production (43–45). In our model, CD4^+^ T cells activated under lipoprotein-deprived conditions or in presence of U18666A exhibited reduced *MAF* mRNA (Figure 4A) and protein levels (Figure 4B), and LDL supplementation partly restored the expression levels (Figure 4, A and B). Similarly, lipoprotein deprivation led to reduced expression of FOXP3 (Supplemental Figure 3, A and B), a key transcription factor involved in IL-10 production and Treg function (41). In the same experimental setup, we measured the mRNA and protein levels of RORγt and T-BET, the main regulators of IL-17 and IFN-γ respectively. *RORC* mRNA and protein levels were reduced in CD4^+^ T cells activated under lipoprotein-deprived conditions or with NPC1 inhibition (Figure 4, C and D). Yet importantly, lipoprotein deprivation had no effect on *TBX21* mRNA and protein (T-BET) expression (Figure 4, E and F), indicating that the effect of this treatment is differential.

As a functional readout for the changes in the levels of the measured transcription factors, we assessed mRNA and protein levels of IL-10, IL-17, and IFN-γ. Reduced c-MAF and FOXP3 expression in CD4^+^ T cells activated under lipoprotein-deprived conditions resulted in lower *IL10* mRNA levels, while *IL17A* and *IFNG* levels did not decrease (Supplemental Figure 3C). The levels of secreted IL-10, IL-17A, and IFN-γ by activated CD4^+^ T cells were reduced under both lipoprotein-deprived condition and NPC1 inhibition, but only IL-10 levels were enhanced by the supplementation of LDL to the lipoprotein-deprived medium (Figure 4G). Thus, LDL uptake by CD4^+^ T cells predominantly affects c-MAF and FOXP3 levels, accompanied by *IL10* mRNA and protein expression. Similar lipoprotein-dependent effects on IL-10 secretion were observed in monocyte-derived macrophages (Supplemental Figure 3D).

Next, we assessed the stability of these markers in CD4^+^ T cells by measuring their protein expression over time using flow cytometry. Although FOXP3 expression declined after 48 hours, c-MAF and IL-10 levels remained relatively stable over time. Lipoprotein deprivation caused a sustained reduction, while LDL supplementation led to a persistent increase in c-MAF and IL-10 expression, indicating that these effects are not attributable to transient T cell activation (Supplemental Figure 3E). We further measured LAG3 expression on c-MAF^+^ CD4^+^ T cells and observed a decrease under lipoprotein-deprived conditions (Supplemental Figure 3F). Given the role of LAG3 in T cell–mediated suppressive function, this reduction indirectly suggests that lipoprotein deprivation may impair the suppressive capacity of the cells.

Finally, we asked which subset of CD4^+^ T cells is responsible for the IL-10 production in our experiments. We found that IL-10 is predominantly produced by c-MAF^+^CD4^+^ T cells (Figure 4H). A smaller fraction of the produced IL-10 was also produced by c-MAF^–^FOXP3^+^ cells (Figure 4H). To further investigate the role of LDL uptake for FOXP3^+^ Treg induction, we cultured CD4^+^ T cells under Treg-polarizing conditions with IL-2 and TGF-β. We observed decreased FOXP3 expression when Tregs were induced under lipoprotein-deprived conditions compared with control conditions. LDL supplementation to the lipoprotein-deprived medium restored the FOXP3 levels (Figure 4I).

Taken together, our data demonstrate that LDL uptake and processing are critical for c-MAF expression, FOXP3 Treg induction, and IL-10 secretion.

### Endolysosomal LDL processing sustains mTORC1 signaling in activated CD4^+^ T cells.

**Figure 5:**
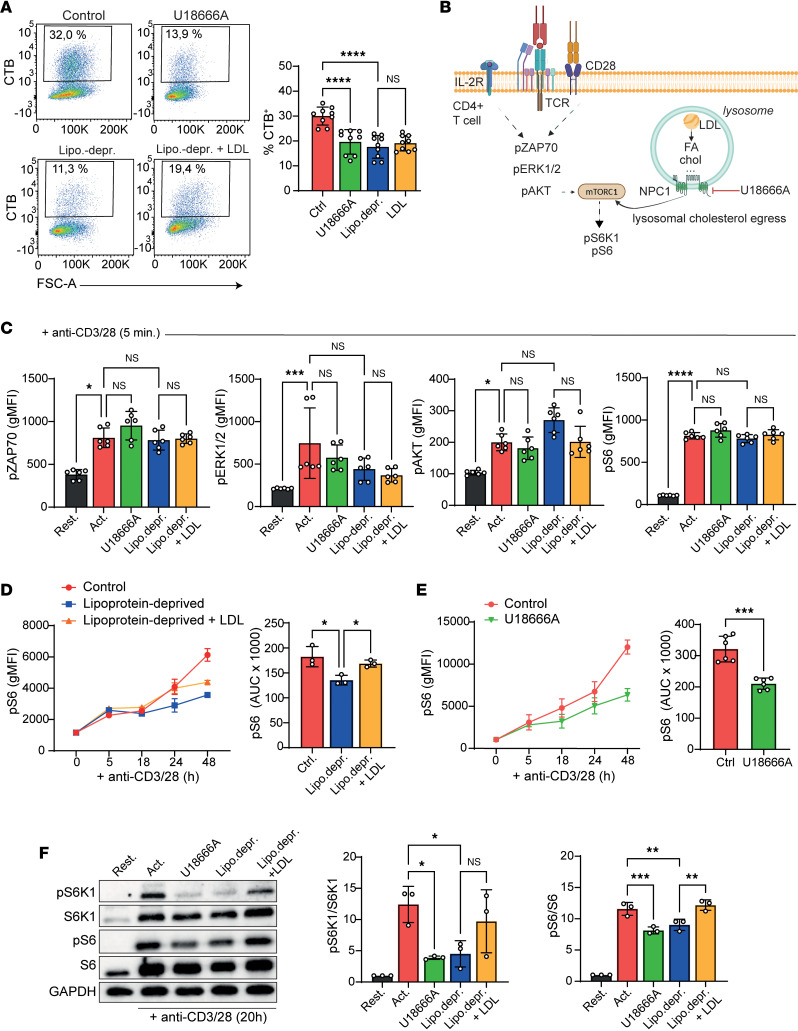
Endolysosomal LDL processing enhances mTORC1 signaling in activated CD4^+^ T cells. (**A**) Flow cytometry staining of lipid microdomains with CTB-FITC. CD4^+^ T cells were activated for 24 hours with anti-CD3/CD28. One-way ANOVA with Dunnett’s multiple comparisons test (****P < 0.0001; *n* = 9). (**B**) Graphical representation of the interplay between TCR signaling and lysosomal LDL processing within CD4^+^ T cells. (**C**) Phospho-flow cytometry of phosphorylated ZAP70 (pZAP70), pERK1/2, pAKT, and pS6 in CD4^+^ T cells treated as indicated. Kruskal-Wallis with Dunn’s multiple comparisons test (*P < 0.05, ***P < 0.001; *n* = 6). (**D**) pS6 measured with flow cytometry in CD4^+^ T cells. The statistical analysis was performed on the area under the curve (AUC). One-way ANOVA with Bonferroni’s multiple comparisons test (*P < 0.05; *n* = 3). (**E**) pS6 measured with flow cytometry on CD4^+^ T cells after the indicated time of activation with or without U18666A (2 μg/mL). Two-tailed unpaired *t* test (***P < 0.001; *n* = 6). (**F**) Western blot analysis of pS6, pS6K1, and GAPDH of cell lysates from CD4^+^ T cells activated for 18 hours with anti-CD3/CD28. One-way ANOVA with Dunnett’s multiple comparisons test (*P < 0.05, **P < 0.01, ***P < 0.001; *n* = 3).

To investigate the regulatory mechanisms linking LDL uptake and metabolic processing to T cell activation and function, we measured key signaling events following TCR stimulation in the same conditions as above (46). Upon early T cell activation, signaling molecules at the plasma membrane are spatially reorganized in lipid microdomains enriched in cholesterol and sphingolipids to facilitate downstream signaling (47). We assessed lipid microdomain formation by staining with FITC-conjugated cholera toxin B (CTB), which binds GM1 in these microdomains, and quantifying fluorescence by flow cytometry. Compared with the control conditions, the CTB signal decreased in CD4^+^ T cells activated under lipoprotein-deprived conditions and with U18666A treatment, indicating disrupted lipid microdomain integrity (Figure 5A). Supplementing LDL to the lipoprotein-deprived medium did not restore the CTB signal, suggesting that LDL alone is insufficient to recover lipid microdomain stability under these conditions.

Next, we considered that the disrupted lipid microdomain integrity in lipoprotein-deprived conditions may impact early TCR signaling (Figure 5B). We therefore assessed early TCR signaling by measuring the phosphorylation of ZAP70, AKT, ERK1/2, and S6, after in vitro CD4^+^ T cell activation (5 minutes) using phospho-flow cytometry. For all experimental conditions the phosphorylation levels were comparable (Figure 5C), indicating no effect on early TCR signaling related to lipoprotein uptake and processing.

As we found that LDLR surface expression was upregulated after 5 hours of CD4^+^ T cell in vitro activation (Figure 1D), LDLR-mediated lipoprotein uptake might have more of an effect on sustaining downstream TCR signaling, rather than an effect on early TCR signaling events. Consistent with this, lysosomal cholesterol availability was proposed as a driver for persistent mTORC1 activation in cell lines, including HEK293T cells and mouse embryonic fibroblasts (26–28). To assess sustained mTORC1 signaling in CD4^+^ T cells, we measured phospho-S6 levels in CD4^+^ T cells activated over time using phospho-flow cytometry. S6 phosphorylation was reduced in CD4^+^ T cells activated under lipoprotein-deprived conditions (Figure 4D). Supplementing with LDL partially restored phospho-S6 to levels comparable to the control condition (Figure 5D). Treatment with U18666A during CD4^+^ T cell activation also led to a decrease in phospho-S6 levels (Figure 5E). To corroborate the observed effects on mTORC1 activity in CD4^+^ T cells, we measured the phosphorylation of mTORC1 targets S6 and S6K1 by immunoblotting. Consistent with the flow cytometry results, immunoblot analysis revealed reduced phosphorylation of S6 and S6K1 under lipoprotein-deprived conditions or with treatment with U18666A (Figure 5F).

Given the observed dependence of mTORC1 activity on lipoprotein availability, we next examined whether mTORC1 signaling regulates expression of c-MAF, FOXP3, and IL-10. Pharmacological inhibition of mTORC1 using either rapamycin or torin led to reduced expression of c-MAF, FOXP3, and IL-10 in activated CD4^+^ T cells (Supplemental Figure 4), indicating that expression of these markers is at least partially dependent on mTORC1 activity.

Together, these results suggest that, while LDL uptake and lysosomal cholesterol egress in activated CD4^+^ T cells do not affect early TCR signaling events, they play a crucial role in maintaining mTORC1 signaling, thereby supporting downstream transcriptional programs associated with T cell function.

### Cells from patients with hoFH confirm the relevance of LDLR for acquiring a c-MAF/IL-10– and FOXP3-driven CD4^+^ T cell phenotype.

**Figure 6:**
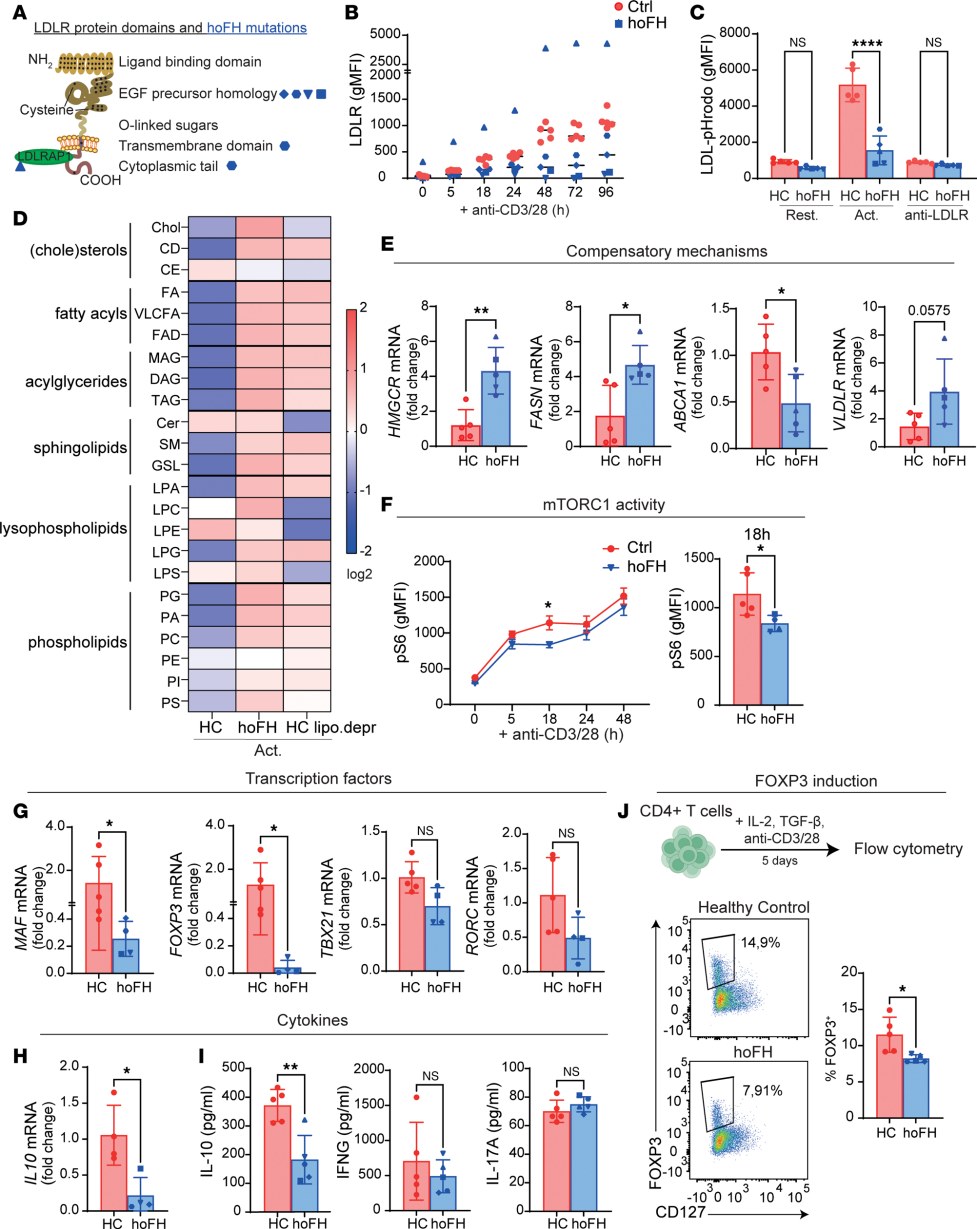
CD4^+^ T cells from patients with hoFH confirm a role of LDLR signaling for CD4^+^ T cell function. (**A**) Schematic of LDLR protein domains and LDLRAP1, highlighting hoFH mutations (blue geometric shapes). (**B**) Cell surface LDLR expression on CD4^+^ T cells from patients with hoFH (*n* = 5) and individuals acting as healthy controls (HC) (*n* = 5). (**C**) Uptake of LDL-pHrodo measured by flow cytometry, where CD4^+^ T cells were activated for 24 hours, cultured for 2 hours in lipoprotein-deprived medium and 2 hours with LDL-pHrodo. Anti-LDLR (5 μg/mL) was added as indicated. One-way ANOVA with Šídák’s multiple comparisons test (****P < 0.0001; *n* = 5 HC and *n* = 5 hoFH). (**D**) Lipidome analysis of CD4^+^ T cell activated for 36 hours. The mean log_2_ values from HC (*n* = 5), hoFH (*n* = 5), and HC in lipoprotein-deprived conditions (*n* = 4) are shown. (**E**) CD4^+^ T cells from HCs (*n* = 5) and patients with hoFH (*n* = 5) were activated in vitro for 15 hours and qPCR was performed to measure the expression *HMGCR*, *FASN*, *ABCA1*, and *VLDLR* genes. Unpaired *t* tests (*P < 0.05, **P < 0.01). (**F**) pS6 measured with flow cytometry in CD4^+^ T cells after the indicated time of activation in CD4^+^ T cells from HC (*n* = 5) or individuals with hoFH (*n* = 4). Mann-Whitney *U* test (*P < 0.05). (**G** and **H**) *MAF*, *FOXP3*, *TBX21*, *RORC*, and *IL10* mRNA levels in CD4^+^ T cells activated for 24 hours. Levels were normalized to RPL13A housekeeping gene and shown in relation to HC. Mann-Whitney *U* test (*P < 0.05; *n* = 5 HC and *n* = 4 hoFH). (**I**) Concentration of secreted cytokines IL-10, IFN-γ, and IL-17A by CD4^+^ T cells activated for 24 hours measured with ELISA. Mann-Whitney *U* tests (**P < 0.01; *n* = 5 HC and *n* = 5 hoFH). (**J**) CD4^+^ T cells were cultured under Treg-polarizing conditions (IL-2 and TGF-β) for 5 days. FOXP3 and CD127 expression was measured by flow cytometry. Mann-Whitney *U* test (*P < 0.05; *n* = 5 HC and *n* = 5 hoFH).

Our experimental model thus far enabled us to study the effects of LDL availability on CD4^+^ T cells proliferation and function. To evaluate the impact of altered LDLR function on CD4^+^ T cell activity in a patient-relevant model, we analyzed CD4^+^ T cells from individuals with hoFH (OMIM 143890 and OMIM 603813) who carry biallelic pathogenic mutations leading to dysfunctional LDLR (Figure 6A and Supplemental Figure 5A). We measured LDLR expression with flow cytometry and confirmed that, compared with those from individuals acting as healthy controls, activated CD4^+^ T cells from individuals carrying *LDLR* mutations exhibited reduced LDLR surface expression (Figure 6B). CD4^+^ T cells from an individual with autosomal recessive hypercholesterolemia, caused by mutations in *LDLR* adaptor protein 1 (*LDLRAP1*), showed increased LDLR surface expression (Figure 6B), which was expected, as mutations in *LDLRAP1* disrupt LDLR internalization, thereby reducing LDL uptake, while maintaining normal or elevated surface occupancy of structurally intact LDLR (48, 49). Next, we evaluated the endolysosomal LDL uptake using the LDL-pHrodo assay. Compared with uptake in cells from individuals acting as healthy controls, we observed impaired LDL uptake specifically in activated CD4^+^ T cells from patients with hoFH (Figure 6C). Treatment with an LDLR-blocking antibody reduced LDL uptake in activated CD4^+^ T cells from individuals acting as healthy controls to levels comparable to those of patients with hoFH (Figure 6C). Considering that LDLR can also bind VLDL particles (50), we observed a corresponding decrease in VLDL uptake in activated CD4^+^ T cells from patients with hoFH compared with individuals acting as healthy controls (Supplemental Figure 5B). These data confirm that activated CD4^+^ T cells from individuals with hoFH have impaired LDLR-mediated lipoprotein uptake.

To investigate how impaired LDLR-mediated lipoprotein uptake affects the intracellular lipid composition of CD4^+^ T cells, we utilized a mass spectrometry–based lipidomic approach. We measured the lipid species in activated CD4^+^ T cells from individuals with hoFH and individuals acting as healthy controls. To determine whether the observed effects result from reduced lipoprotein uptake, we included healthy control cells activated under lipoprotein-deprived conditions. Our analysis revealed an overall increase in cholesterol, fatty acyls, acylglycerides, sphingolipids, and (lyso)phospholipid subclasses in CD4^+^ T cells from patients with hoFH compared with individuals acting as healthy controls. Similarly, most lipid classes were elevated in CD4^+^ T cells from individuals acting as healthy controls cultured under lipoprotein-deprived conditions (Figure 6D). This suggests that CD4^+^ T cells compensate for the lack of LDLR-dependent lipoprotein uptake. In support, hoFH patient CD4^+^ T cells showed increased intracellular mevalonate levels (Supplemental Figure 5C) and *HMGCR* levels (Figure 6E) compared with healthy control cells after in vitro activation, suggesting an enhanced flux through the mevalonate pathway. In addition, we observed increased expression of *FASN*, a known SREBP1 target, indicating broader activation of lipid biosynthesis programs. Conversely, expression of *ABCA1*, a key mediator of cellular cholesterol efflux, was reduced in CD4^+^ T cells from patients with hoFH. We also observed a trend of increased *VLDLR* expression in the patients with hoFH (Figure 6E). Together, these data suggest that while LDLR-dependent lipoprotein uptake is compromised in patients with hoFH, compensatory mechanisms enhancing intracellular cholesterol levels are active.

Phenotypic analysis of circulating CD4^+^ T cells from patients with hoFH and individuals acting as healthy controls revealed no differences in the frequency of CD4^+^ T cells, memory subsets or FOXP3^+^ Tregs, or in the ex vivo expression of exhaustion and activation markers (Supplemental Figure 5, D–G). Next, we measured phosphorylation of S6, as a proxy for mTORC1 activity. In vitro activated CD4^+^ T cells from individuals with hoFH exhibited overall decreased phosphorylation of S6 compared with those from individuals acting as healthy controls (Figure 6F). The observed reduction in S6 phosphorylation was statistically significant only after 18 hours of activation. Despite reduced mTORC1 signaling, CD4^+^ T cells from patients with hoFH exhibited unaffected proliferation and activation marker expression upon in vitro activation (Supplemental Figure 5, H and I).

Next, we asked whether CD4^+^ T cells from patients with hoFH exhibit functional differences in response to activation, similar to those observed under lipoprotein deprivation conditions. We observed a reduction in *MAF* and *FOXP3* mRNA levels in activated CD4^+^ T cells from patients with hoFH compared with those from individuals acting as healthy controls (Figure 6G). There were no significant differences in *TBX21* and *RORC* mRNA expression between cells from individuals with hoFH and those acting as healthy controls (Figure 6G). Consistent with the reduced *MAF* and *FOXP3* mRNA levels, *IL10* mRNA expression and IL-10 secretion were lower in activated CD4^+^ T cells from patients with hoFH compared with those from healthy controls (Figure 6, H and I). Secretion of IFN-γ and IL-17A remained unaffected in this setting (Figure 6I). Finally, we explored whether the decline in *FOXP3* levels in in vitro activated CD4^+^ T cells stemmed from overall reduced activation or impaired Treg induction. For this, we cultured CD4^+^ T cells under Treg-polarizing conditions with IL-2 and TGF-β. Cells from individuals with hoFH exhibited reduced Treg induction, evidenced by a lower percentage of FOXP3^+^CD4^+^ T cells (Figure 6J).

Taken together, our data from patients with hoFH confirm that LDLR-mediated lipoprotein uptake is critical for CD4^+^ T cell function, particularly in maintaining mTORC1 signaling, enhancing *MAF* expression and IL-10 production, and facilitating in vitro *FOXP3* expression and Treg induction.

## Discussion

Over the last decade, lipids have emerged as key regulators of T cell function (7). While transcriptional regulators such as SREBP-2 and LXR are well established in coordinating lipid metabolism and T cell function, here we extend this understanding by demonstrating a role for LDLR-mediated lipoprotein uptake in human CD4^+^ T cells (42, 51–56). Most studies to date have focused on CD8^+^ T cells, which tend to accumulate more cholesterol than CD4^+^ T cells, and these studies identified lipoprotein metabolism as a key determinant of CD8^+^ T cell activation and function (22, 57–59). An earlier study employing LDLR-deficient mice suggested a minimal effect on CD4^+^ T cell function, while significant impairment was observed in CD8^+^ T cells (22). However, translating these findings to humans is complicated due to differences in lipoprotein metabolism between species. For example, LDL is the dominant cholesterol carrier in humans, while HDL predominates in mice because they lack cholesteryl ester transfer protein (CETP) (60). These differences suggest that LDLR deficiency could effect human T cell function more than in mice. Here, we demonstrate that LDLR-mediated lipoprotein uptake and metabolism is active and indeed functionally relevant in human CD4^+^ T cells.

Cholesterol is an essential component of cellular membranes, contributing to membrane fluidity and the formation of lipid microdomains that support TCR clustering and efficient signal transduction during T cell activation (61). In a recent study, Jacobs et al. reported that decreased LDLR surface abundance in T cells from patients with chronic lymphocytic leukemia was associated with impaired lipid microdomain formation and reduced activation-marker expression compared with healthy control T cells (10). In line with their findings, we observed reduced surface expression of activation markers and diminished proliferation in CD4^+^ T cells from healthy individuals when cultured in lipoprotein-deprived medium. However, T cells from patients with a genetic impairment in LDLR-mediated LDL uptake (patients with hoFH) did not exhibit reduced proliferation or activation. This suggests that LDLR-independent lipid acquisition pathways may compensate for defective LDLR-mediated uptake in patients with hoFH, at least in terms of supporting T cell activation and proliferation. Indeed, we observed increased intracellular lipid subsets, including mevalonate and cholesterol, in CD4^+^ T cells from patients with hoFH. Moreover, increased *HMGCR* expression supports enhanced de novo cholesterol biosynthesis as a compensatory mechanism. We observed a trend toward increased *VLDLR* expression, suggesting that receptor redundancy may play a more limited role compared with cholesterol biosynthesis in CD4^+^ T cells. Together, these findings indicate that both lipoprotein uptake and cholesterol biosynthesis are required to sustain CD4^+^ T cell activation and proliferation in healthy control CD4^+^ T cells, yet CD4^+^ T cells from patients with hoFH are relatively efficient at compensating for the loss of LDLR-mediated lipoprotein uptake.

We revealed a critical role of LDLR-mediated lipoprotein uptake for expression and secretion of the immune regulatory cytokine IL-10 by in vitro activated CD4^+^ T cells. In line with our experimental data, studies in patients with hoFH have shown decreased in vivo IL-10 levels (62) and an overall proinflammatory phenotype (63, 64). The proinflammatory phenotype of patients with hoFH may, at least in part, stem from the decreased capacity of their CD4^+^ T cells to produce IL-10. Nonetheless, to the best of our knowledge, patients with hoFH do not show enhanced autoimmunity. Interestingly, hypercholesterolemia itself has been shown to enhance TCR signaling, CD4^+^ T cell proliferation, and FOXP3^+^ Treg development, indicating that systemic cholesterol levels can directly influence T cell activation and polarization (65). The number of patients with hoFH available for our studies was limited, and future studies in larger cohorts should further establish any immune phenotypes associated with hoFH. Our results show that LDL-derived cholesterol is needed for the expression of c-MAF, a master regulator of IL-10 in immune cells (41). Previous studies have identified cholesterol as a critical regulator of c-MAF and IL-10 in CD4^+^ T cells (42) and B cells (66). Specifically, in B cells geranylgeranyl pyrophosphate, an intermediate in the cholesterol biosynthesis pathway, was identified as the main regulator of c-MAF(66). In CD4^+^ T cells, however, supplementation with geranylgeranyl pyrophosphate under conditions of inhibited cholesterol biosynthesis failed to restore c-MAF expression, and the authors did not identify the molecular pathways involved (42). In our experiments, LDLR-deficient CD4^+^ T cells exhibit active cholesterol biosynthesis but have a reduced capacity to internalize and process LDL cholesterol. Therefore, cholesterol derived from LDL internalization may be a critical regulator of c-MAF and IL-10 levels. LDL deprivation also reduced expression of additional transcription factors, including FOXP3 and RORγt, suggesting broader effects of LDLR-dependent cholesterol uptake on CD4^+^ T cell differentiation programs. However, the molecular mechanisms linking LDL-derived cholesterol to regulation of these transcription factors and suppressive capacity of CD4^+^ T cells remain unclear and warrant further investigation. Notably, FOXP3 and c-MAF act cooperatively, but are regulated independently, as genetic deletion of c-MAF does not impair FOXP3 expression or Treg development, and c-MAF can be induced in FOXP3^–^ T cells without altering FOXP3 levels, arguing against a direct regulatory relationship between these transcription factors (67). However, both partly depend on mTORC1 activity.

In macrophages, inhibition of mTORC1 is known to decrease c-MAF expression, suggesting that mTORC1 signaling could also provide a link between LDL uptake and c-MAF expression in CD4^+^ T cells (68). Lysosomal cholesterol availability can influence mTORC1 activation, a key regulator of T cell metabolism, growth, and differentiation (69). Mechanistically, mTORC1 activity is regulated at the lysosomal surface, where the complex is recruited by heterodimers of the Rag family of small GTPases (69). A previous study showed that depleting HEK293T cells of LDL-derived cholesterol downregulated mTORC1 and disrupts its lysosomal localization (27). Thus far, two pathways involving the lysosomal amino acid transporter SLC38A9 and the lysosomal sensing protein LYCHOS have been implicated in lysosomal cholesterol sensing and subsequent Rag activity (27, 28). However, it remains unknown whether these cholesterol sensing pathways are active in immune cells. Consistent with our findings, Bonacina et al. have reported that LDLR-deficient mouse CD8^+^ T cells also exhibit reduced mTORC1 activity (22). Furthermore, we showed that NPC1 inhibition reduced mTORC1 signaling in activated CD4^+^ T cells. NPC1 is a well-characterized lysosomal cholesterol exporter, and we reasoned that its inhibition would limit the lysosomal cholesterol egress needed for mTORC1 activation (70). However, the role of NPC1 in mTORC1 activation may vary between cell types. Previous studies in the nonimmune cell lines HEK293T and mouse embryonic fibroblasts identified NPC1 as a negative regulator of mTORC1 (26, 27). These cell lines have constitutively active mTORC1, which makes them different from primary human T cells, where mTORC1 is only active upon cellular activation. We therefore propose that NPC1 inhibition in T cells causes lysosomal cholesterol accumulation at the expense of plasma membrane and ER cholesterol pools, thereby weakening sustained TCR signaling and reducing overall mTORC1 activity despite preserved lysosomal cholesterol-dependent Rag signaling. Further work is needed to define NPC1-dependent and -independent mechanisms regulating mTORC1 activation by lysosomal cholesterol in human CD4^+^ T cells.

**Figure 7:**
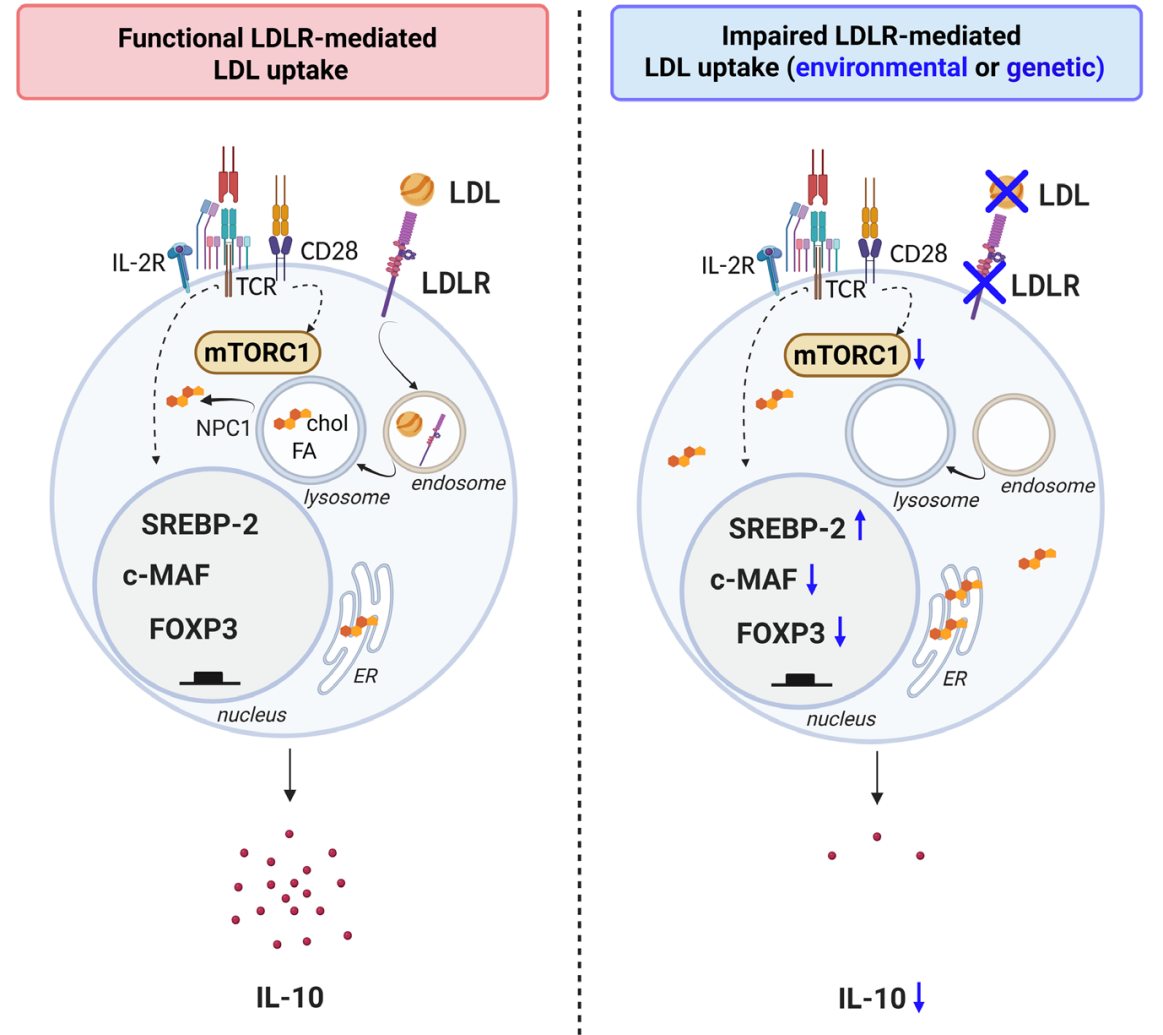
Schematic representation of the proposed model depicting CD4^+^ T cell activation under conditions of functional versus impaired LDLR-mediated LDL uptake. Upon TCR-driven activation (anti-CD3/CD28 Dynabeads in vitro), CD4^+^ T cells upregulate LDLR expression and LDL uptake. When LDLR-mediated lipoprotein uptake is impaired, due to lack of LDL in the extracellular environment or genetic mutations in the *LDLR* (blue in the scheme), CD4^+^ T cell function is hampered. First, SREBP-2–driven cholesterol synthesis is upregulated as a compensatory mechanism to counteract the reduced LDLR-mediated lipoprotein uptake. Second, mTORC1 activity decreases upon in vitro CD4^+^ T cell activation under both environmental and genetic impairment of LDLR-mediated lipoprotein uptake. Finally, c-MAF and FOXP3 levels are decreased, leading to reduced secretion of the immune regulatory cytokine IL-10.

Taken together, we propose that LDLR-mediated LDL uptake sustains mTORC1 activity, thereby promoting c-MAF expression and IL-10 production by CD4^+^ T cells activated by TCR engagement (Figure 7). Our finding that LDLR-mediated lipoprotein uptake by CD4^+^ T cells affects immunological function may have translational implications. Theoretically, lipid-lowering drugs such as PCSK9 inhibitors could be harnessed to modulate lipoprotein uptake and change T cell function (71). PCSK9 inhibitors enhance LDLR surface expression by promoting receptor recycling and are routinely used to lower plasma LDL levels (72). In atherosclerosis for example, PCSK9 functions extend beyond regulating plasma LDL cholesterol levels and have been implicated in modulating inflammation within the adaptive immune system (72). However, harnessing LDLR-mediated lipoprotein uptake by CD4^+^ T cells for immunomodulation is not straightforward, as CD4^+^ T cells only need small amounts of lipoproteins to fulfill their nutritional needs upon activation and seem to engage alternative uptake and lipid biosynthesis mechanisms.

## Methods

### Sex as biological variable.

There was no exclusion of participants on the basis of sex in this study, and both female and male participants were included.

### Cell purification and culture.

Peripheral blood from healthy individuals and 5 patients with hoFH was collected into BD Vacutainer blood collection tubes with sodium heparin. PBMCs were isolated with Ficoll-Paque density gradient centrifugation. CD4^+^ T cells were isolated from full PBMCs using the CD4 T cell isolation kit (Miltenyi) with autoMACS (Miltenyi) automated cell isolation following the manufacturer’s protocol. CD4^+^ T cells were cultured in RPMI 1640 (Thermo Fisher Scientific) supplemented with 10% heat-inactivated human serum, 1% penicillin-streptomycin, and 2 μM L-glutamine. To activate the CD4^+^ T cells, human T cell activator CD3/CD28 Dynabeads (Gibco) were added in a ratio of beads to CD4^+^ T cells of 1:5, as well as recombinant human IL-2 (Novartis; 10 IU/mL). Monocyte-derived macrophage cultures were performed as previously published (73). HEK293T cells (CRL-3216) were cultured in DMEM (Thermo Fisher Scientific) supplemented with 1% FBS and 1% penicillin-streptomycin.

### Quantitative real-time PCR.

Total RNA was isolated from the cells using the RNeasy mini kit (Qiagen) according to the manufacturer’s protocol. For each sample, an equal amount of RNA was transcribed into cDNA with the cDNA Synthesis Kit (Bio-Rad). To perform the quantitative real-time PCR (qRT-PCR) reaction we used SYBR green qPCR master mix (Bio-Rad) and primers that are shown in Supplemental Table 1. Measurements were done with QuantStudio 3 (Fisher Scientific), and gene expression levels were determined according to the ΔCt methods (relative abundance = 2^(–Δ**Ct**)^). *RPL13A* was used as housekeeping gene.

### Flow cytometry.

Cells were washed with PBS and stained with Fixable Viability dye eFluor 507 (eBioscience) for 20 minutes at 4°C to identify and exclude the dead cells from the analysis. The cells were then washed with FACS buffer (PBS supplemented with 2% FCS and 0.1% sodium azide) followed by a blocking incubation with 10% mouse serum in FACS buffer. The cells were then stained with surface antibodies for 20 minutes at 4°C. For intracellular staining, the cells were fixed and permeabilized using Fixation and Permeabilization buffer (eBioscience) for 30 minutes at 4°C. For detection of phosphorylated ZAP70 (pZAP70), pAKT, pERK1/2, and pS6, phospho-flow was used, and the cells were fixed with BD Phosflow Fix buffer I and permeabilized with BD Phosflow Perm buffer III. The cells were then stained with the antibodies against the intracellular targets for 30 minutes at 4°C. The antibodies used are listed in Supplemental Table 2, and flow cytometry negative control staining is shown in Supplemental Figure 6. The cells were washed twice in FACS buffer and measured on the BD LSR Fortessa with FACSDiva software. Analysis was performed with FLowJo (V.10.5.3).

### Western blotting.

The cells were washed in ice-cold PBS and lysed in RIPA buffer (Sigma) in presence of Halt Protease Inhibitor Cocktail (Thermo Fisher) followed by boiling (10 minutes at 95°C). The lysates were then centrifuged, and the supernatant was collected in new tubes. Protein concentration was measured with the BCA protein assay kit (Pierce). Samples were then mixed with Laemmli loading buffer. An equal amount of protein was run on SDS-PAGE and transferred onto polyvinylidene fluoride membrane (Merck Millipore). We blocked the membranes with 5% milk powder (Campina) in PBS 0.1% Tween-20 for 1 hour before probing with the specific primary antibodies for 16 hours at 4°C. Subsequently, the membranes were washed and stained with HRP-coupled secondary antibodies for 1 hour at room temperature. The membranes were again washed with PBS 0.1% Tween-20. We analyzed the images on ChemiDoc Imaging System (Bio-Rad). The antibodies used were anti-LDLR (R&D Systems), anti-S6 ribosomal protein (Bioke), anti-phospho-S6 Ribosomal Protein (Ser240/244) (Bioke), anti-p70 S6 Kinase (Bioke), anti-phospho-p70 S6 Kinase (Thr389) (Bioke), and anti-GAPDH (AntibodyChain International B.V.).

### Lipoprotein isolation and fluorescence labeling.

Lipoproteins were isolated from human serum (Sanquin) using a previously established ultracentrifugation protocol with KBr gradient (105,000 *g* for 20 hours with breaks off) (74). Isolated lipoproteins were fluorescently labeled either with DyLight 633 NHS Ester (Thermo Fisher Scientific) or pHrodo iFL Green STP Ester (amine-reactive) (Thermo Fisher Scientific), according to the supplier’s instructions.

### CellTrace Violet proliferation assay.

For the proliferation analysis, CD4^+^ T cells were resuspended in PBS (1 million/mL) and stained with CellTrace Violet (Thermo Fisher Scientific, 2.5 μM) for 30 minutes at room temperature. The staining was stopped by adding 3 times the volume of culture medium. The cells were then washed and resuspended in culture medium with or without lipoproteins, as indicated in the experimental description. The cells were cultured for 4 days and analyzed by flow cytometry. The data were analyzed using the proliferation tool in FLowJo (V.10.5.3). The proliferation index, as the sum of the cells in all generations divided by the computed number of original parent cells present at the start of the experiment, was plotted.

### Confocal microscopy.

For intracellular visualization of SREBP-2, in vitro activated (24 hours) CD4^+^ T cells were fixed with 4% PFA for 15 minutes at 4°C. Cells were then permeabilized with 0.1% Triton X-100 (Merk) in 0.1% PBSA (bovine serum albumin dissolved in PBS) for 10 minutes at room temperature. Subsequently, the cells were blocked with 10% normal goat serum in 1% PBSA for 1 hour at room temperature. The cells were then stained with primary antibody (goat anti-SREBP-2; R&D Systems; 10 μg/mL) for 3 hours at room temperature. The cells were then washed in 1% PBSA and stained with donkey anti-goat IgG (H+L) cross-adsorbed secondary antibody and Alexa Fluor 488 (Life Technologies) diluted 1:400 in 1% PBSA for 1 hour at room temperature. Then, the cells were washed (with PBS), and the nuclei were stained with Hoechst (1:50,000 dilution at room temperature for 20 minutes). Finally, the cells were washed with PBS and visualized and acquired on a Stellaris 5 Leica confocal microscope using LAS X software, before images taken at ×63 magnification (oil). The signal was quantified using CellProfiler 4.2.5.

### Detection of secreted cytokines.

CD4^+^ T cell culture supernatants were collected and stored at –80°C until analyzed. Cytokine levels were measured using specific ELISA kits for human IFN-γ, IL-10, IL-17A and PCSK9 (BioLegend) following manufacturer’s protocol. Calorimetric measurements were performed using CLARIOstar ELISA plate reader (BMG Labtech).

### Liquid chromatography mass spectrometry.

Cell samples in 80% ice-cold methanol were evaporated to dryness in a Centrivap sample concentrator at 40°C. To the dry samples 10 μL internal standards mixture, 190 μL methanol, and 400 μL chloroform were added. The samples were homogenized by ultrasonification for 5 minutes and incubated for 1 hour at 30°C and 900 rpm in a thermoshaker. Phase separation was induced by adding 200 μL water. The samples were pulse vortex mixed, stored for 10 minutes at 4°C, and centrifugated for 10 minutes at 4°C and 1717,000 × *g*. Both the upper aqueous phase and lower organic phase were collected and evaporated to dryness in a Centrivap concentrator. The organic phase was dissolved in 100 μL acetonitrile and analyzed using a Thermo Scientific Vanguard Quaternary UHPLC system with an Acquity BEH C18 column (2.1 × 100 mm, 1.7 μm) coupled to an Exploris 480 Orbitrap MS equipped with a HESI source operated in negative (– 2.5kV) and positive (3.6 kV) mode. The system was operated at a flow rate of 450 μL/min and a column temperature of 60°C. The mobile phases consisted of solvent A (40% acetonitrile containing 10 mM ammonium acetate) and solvent B (10% acetonitrile and 90% isopropanol, also containing 10 mM ammonium acetate) for both negative and positive ion modes. A 12-minute linear gradient of 40%– 100% B was started after the injection of 5 μL of the sample. The system was kept at 100% B for the next 5 minutes, after which the system returned to its starting situation. Total runtime was 20 minutes. Data analysis was performed using MZMine 2 open source software (75). The isolated aqueous phase was dissolved in ultrapure water and analyzed in the same liquid chromatography mass spectrometry setup equipped with an Atlantis Premier C18AX column (2.1 × 100 mm, 1.7 μm). Sample analysis was conducted at high and low pH at 30°C and a flow rate of 300 μL/min in both ionization modes. Mobile phase A at high pH consisted of 20 mM ammonium acetate and 5 mM ammonium hydroxide, and at low pH it consisted of 0.1% formic acid. Mobile phase B was acetonitrile in both cases. A step gradient was started upon injection of 5 μL of sample. The gradient steps were 0% B for 1.5 minutes followed by a 5-minute linear increase to 15% B, after which the percentage B was increased linearly to 70% over 3.5 minutes. The gradient was kept at 70% for 2 minutes, after which the system returned to 0% B in 0.2 minutes, and the column was allowed to reequilibrate for 7 minutes prior to a next injection. Targeted data analysis was performed using XCalibur Quan Browser v4.7 software.

### RNA-seq data.

CD4^+^ T cells were lysed in TRIzol LS reagent (Thermo Fisher), and RNA was isolated using the Dynabead mRNA Purification Kit (Thermo Fisher) according to the manufacturer’s protocol. Polyadenylated messenger RNA was isolated using Poly(A) beads (NEXTflex), and sequencing libraries were made using the Rapid Directional RNA-seq kit (NEXTflex). Libraries were sequenced at the Utrecht Sequencing Facility using the Nextseq500 platform (Illumina), which produced single-end reads of 75 bp. Reads were trimmed using *Trim Galore* v0.6.10 with default parameters and aligned to the reference genome (GENCODE: release v46 [GRCh38.p14], primary assembly) using *STAR* v2.7.11b42 in 2-pass mode basic and *--quantMode* to count reads per gene using *htseq-count*. Genes with at least a sum of 10 reads in the minimum of samples per group were selected to continue the analysis, and *biomart* v.2.60.143 was used for gene annotation. Read counts were normalized using *DESeq2* v1.44.0^44^ with the model *~ Disease + Sex*, which accounts for sex effects when estimating disease-specific differences. The *results* function of *DESeq* was used to identify differentially expressed genes, and *P* values were adjusted for multiple testing using the FDR. Log_2_ fold change values were shrunken using the default and recommended *apeglm* v1.26.1^45^ algorithm of the *lfcShrink* function. For the synovial fluid oligo JIA data, differential expression results were obtained from Mijnheer et al. (76), and expression differences belonging to the canonical isoform or, if not available, the isoform with higher mean normalized read counts (*baseMean*) were selected per gene. Genes with an adjusted *P* value of <0.05 and at least double difference of expression between conditions (absolute log_2_FC >1) were considered as differentially expressed. Functional enrichment analysis was performed for overexpressed genes using *gprofiler*2 v0.2.3^46^ correcting for multiple testing by FDR, and significance was considered with an adjusted *P* value <0.05. *pathview* v1.44.0 was used to show gene expression for selected KEGG pathways, and *rrvgo* v1.16 was used to reduce (default parameters and threshold = 0.7) and visualize enriched Gene Ontologies of Biological Process.

### Statistics.

The Shapiro-Wilk test was used to assess normality of the data. Statistical significance between 2 groups was determined using 2-tailed unpaired *t* tests for parametric data and Mann-Whitney *U* tests for nonparametric data. When comparing multiple groups, 1-way ANOVA and Kruskal-Wallis test were used. The analyses were performed using GraphPad Prism (V.8.3.0). A *P* value of less than 0.05 was considered significant. Data represent mean ± SD.

### Study approval.

Ethical approval for the collection of blood from healthy volunteers was obtained from the Institutional Ethical Review Board of the University Medical Center Utrecht (protocol 07-125/C). Ethical approval for inclusion of 5 patients with hoFH was obtained from the Medical Research Ethics Committee of the Erasmus Medical Center Rotterdam (protocol MEC-2023-0742; the study complies with the Declaration of Helsinki). Written informed consent was obtained from all participants.

### Data availability.

All data in the article are included in the Supporting Data Values file and supplemental materials. The raw RNA-seq data from the cohort are not publicly available to protect research participant privacy and due to lack of consent for sharing these data. Raw data can be accessed via DataverseNL. There are restrictions on use by commercial parties and on sharing openly based on (inter)national laws and regulations and the written informed consent. Therefore these data are only available upon discussion and signing a data sharing agreement (see Terms of Access in DataverseNL) and within a specially designed UMC Utrecht provided environment. Additional information can be provided by the corresponding author upon request.

## Author contributions

AM, HSS, and MB initiated the study and wrote the manuscript. AM performed the experiments and analyzed the data. NSVH assisted with microscopy experiments. DD and GR assisted with experimental work during the revision process. ECAS and CG performed mass spectrometry experiments and analyzed the data. AB performed the RNAseq analysis. LVDZ, MTM, and JERVL assisted in obtaining PBMCs from patients with hoFH. EM, JvL, NZ, PCNR, SK, and SJV contributed to substantial discussions and provided feedback throughout the project.

## Conflict of interest

The authors have declared that no conflict of interest exists.

## Funding support

- Actuate Therapeutics to MB.
- Netherlands Organization for Scientific Research (NWO) Veni grant (91618150) to HSS.
- Erasmus MC Research Fellowship and Starting Grant to HSS.
- NWO Vici grant (016.176.643) to NZ.
- NWO Domain Science (ENW) grant (M.22.034; GENESIS) to NZ.
- Sobi unrestricted research grant in the context of a public-private grant mechanism (together with ZonMW) to SJV.
- Finances for an investigator-initiated grant about shared decision making for the choice of PCSK9 inhibitors (Novartis) to JERVL’s department.

## Supplementary Material

Supplemental data

Unedited blot and gel images

Supporting data values

## Version 1 06/08/2026

Electronic publication
