# Supplementary material for: LDL receptor–mediated lipoprotein uptake fuels human CD4+ T cell polarization toward a c-MAF/IL-10– and FOXP3-driven phenotype
Source: JCI Insight. 2026 Jun 8;11(11):e198505. doi: 10.1172/jci.insight.198505 (PMC13313490; doi:10.1172/jci.insight.198505)
Supplement: Supplemental data [file jciinsight-11-198505-s115.pdf]

LDL receptor-mediated uptake of lipoproteins fuels human CD4+ T cell polarization towards a c-MAF-IL-10 and FOXP3-driven regulatory phenotype

Angela Markovska[1,2], Niels S. van Heusden[1], Dagmar D. Duijzer[1], Alejandra Bodelón[1], Greta Rogani[1],Enric Mocholi[3,4], Edwin C.A. Stigter[5], M. Can Gulersonmez[5], Sander Kooijman[6], Leonie van der Zee[7], Monique T. Mulder[7], Jeanine E. Roeters van Lennep[7], Patrick C.N. Rensen[6], Jorg van Loosdregt[1], Sebastiaan J. Vastert[1,8], Noam Zelcer[9, 10, 11], Marianne Boes[1,12*], Henk S. Schipper[1,13*]

1. Center for Translational Immunology, University Medical Centre Utrecht, Utrecht, the Netherlands.
2. Department of Biomolecular Health Sciences, Faculty of Veterinary Medicine, Utrecht University, Utrecht, The Netherlands
3. Center for Molecular Medicine, University Medical Center Utrecht, Utrecht, the Netherlands; Regenerative Medicine Center, University Medical Center Utrecht, Utrecht, the Netherlands.
4. Dep. Ciencias Biomédicas, Universidad Cardenal Herrera CEU, Valencia, Spain
5. Center for Molecular Medicine, University Medical Center Utrecht, Utrecht, Netherlands; Oncode Institute, Utrecht, the Netherlands
6. Department of Medicine, Division of Endocrinology, and Einthoven Laboratory for Experimental Vascular Medicine, Leiden University Medical Center, Leiden, the Netherlands.
7. Department of Internal Medicine Laboratory, Erasmus MC Cardiovascular Institute. Erasmus MC, University Medical Center Rotterdam, Rotterdam, the Netherlands.
8. Department of Pediatric Rheumatology and Immunology, University Medical Center Utrecht, Utrecht, the Netherlands.
9. Department of Medical Biochemistry, Amsterdam UMC location AMC, University of Amsterdam, Amsterdam, the Netherlands.
10. Amsterdam Gastroenterology, Endocrinology, and Metabolism (AGEM) Institute, Amsterdam UMC, Amsterdam, the Netherlands
11. Amsterdam Cardiovascular Sciences (ACS) Institute, Amsterdam UMC, Amsterdam, the Netherlands
12. Pediatrics Department, University Medical Center, Utrecht, the Netherlands.
13. Department of Pediatric Cardiology, Sophia Children's Hospital, Erasmus Medical Center, Rotterdam, the Netherlands.

*authors contributed equally

Corresponding author: Henk S. Schipper, email: h.schipper@erasmusmc.nl

SUPPLEMENTARY INFORMATION

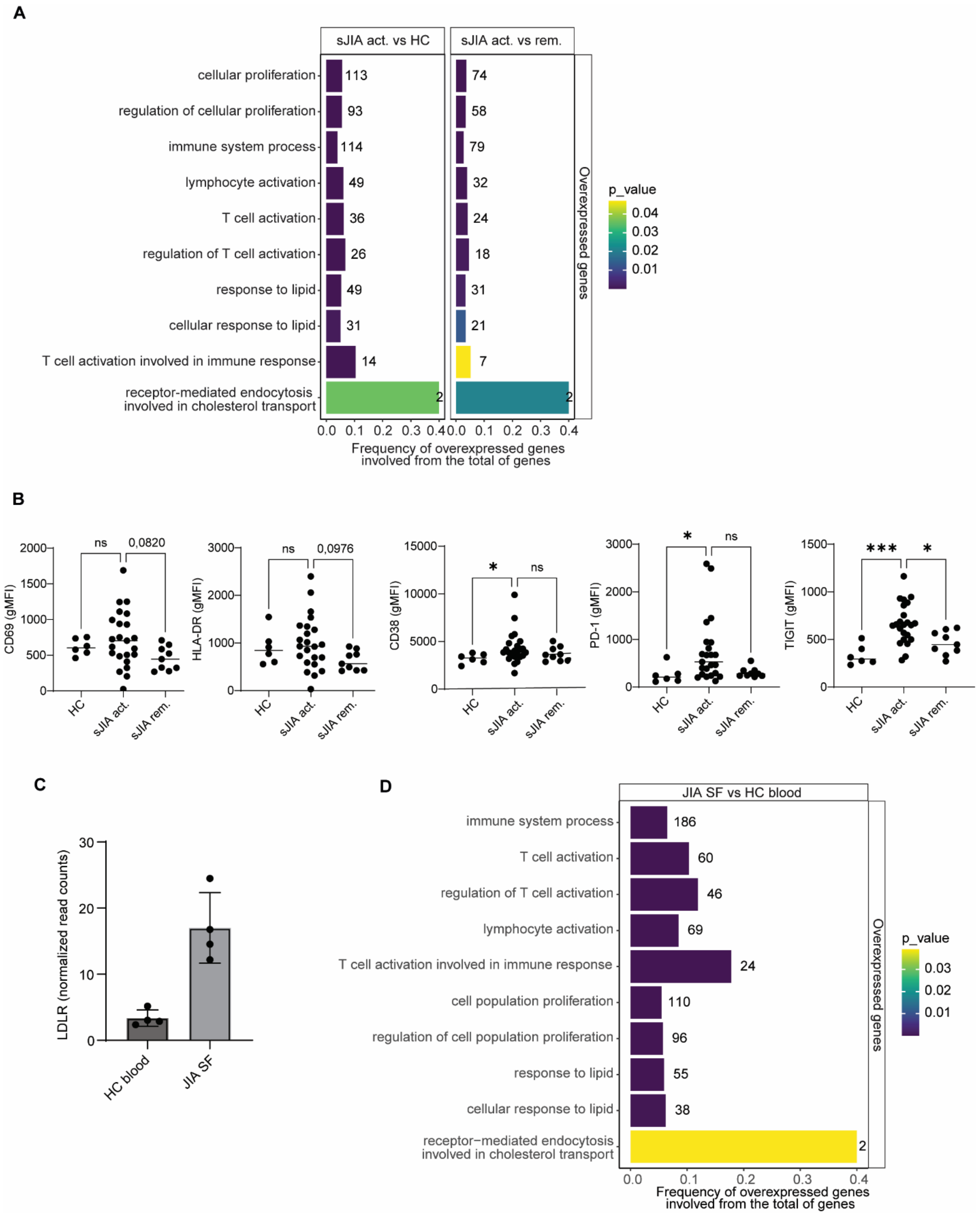

Figure 1. (**A**) Significant Enriched Gene Ontologies of Biological Process (GO:BP) involved in immune activation, cell proliferation, T cell activation and cholesterol transport in active systemic JIA patients vs. healthy controls (left column) or patients in remission (right column) (n=7 per group). The number of overexpressed genes associated with each GO term is shown, along with the proportion they represent of the total genes involved in that pathway. (**B**)

Activation marker expression on CD4+ T cells from healthy controls, patients with active systemic JIA (sJIA) and patients in remission. Activation markers measured using flow cytometry. Mann Whitney test or Kruskal Wallis with Dunn's multiple comparison's test. (**C**) LDLR gene expression in CD4+ oligo JIA synovial fluid (SF) vs heathy adults peripheral blood (PB) (n=4 per group). (**D**) Significant Enriched Gene Ontologies of Biological Process (GO:BP) involved in immune activation, cell proliferation, T cell activation and cholesterol transport for oligo JIA synovial fluid (SF) vs heathy adults peripheral blood (PB) (n=4 per group). The number of overexpressed genes associated with each GO term is shown, along with the proportion they represent of the total genes involved in that pathway.

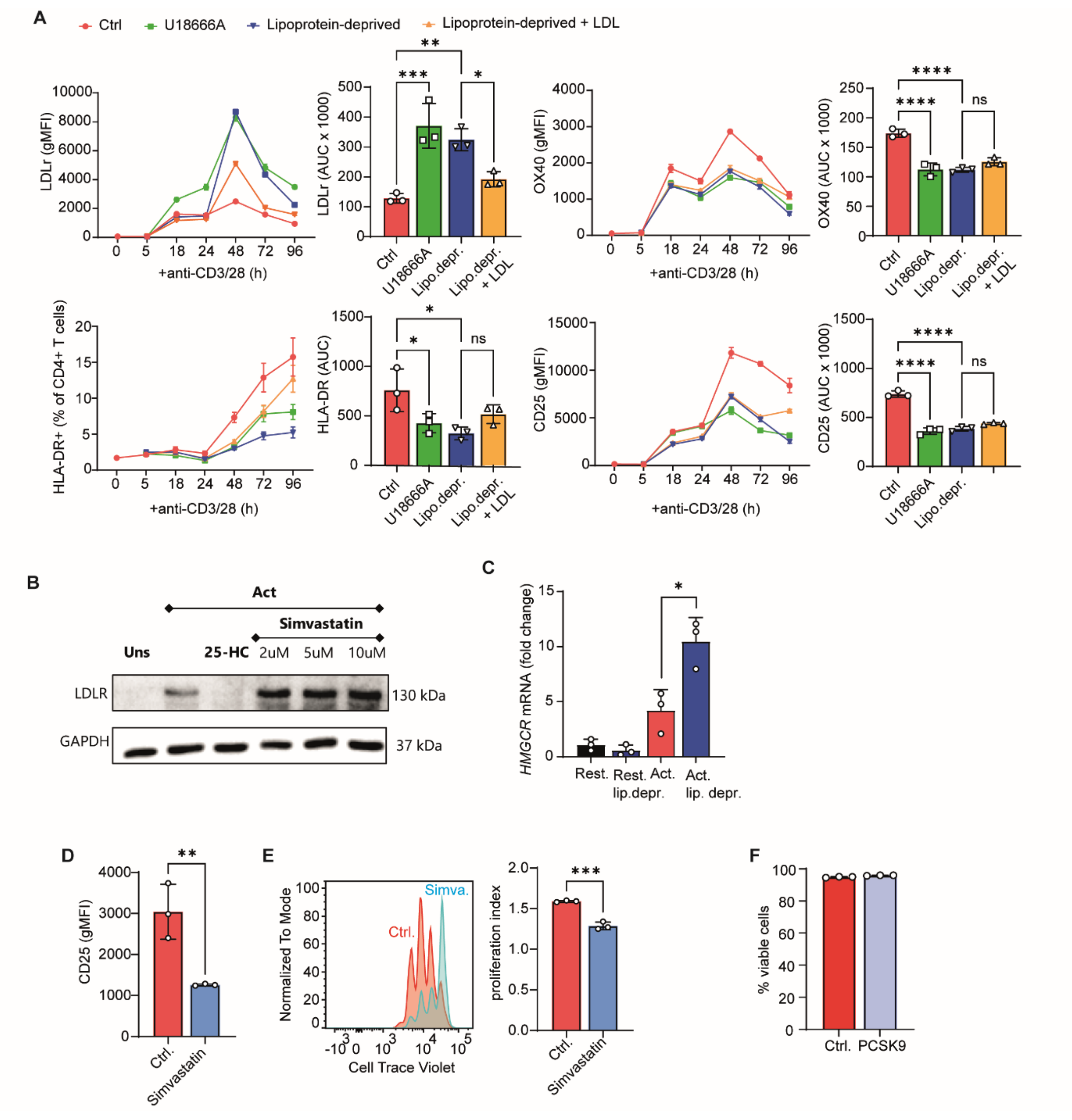

Figure 2. Lipoproteins fuel CD4+ T cell activation and proliferation. CD4+ T cells were activated with anti-CD3/28 Dynabeads and cultured in control (ctrl) medium supplemented with human serum, in lipoprotein-deprived serum

or in lipoprotein-deprived serum supplemented with LDL (10 µg/ml). Where indicated, cells were treated with U18666A (2 µg/ml) (**A**) Cell surface expression of LDLR, OX40, HLA-DR and CD25 measured on flow cytometry. The statistical analysis is done on the area under the curve (AUC). One-way ANOVA with Šídák's multiple comparisons test, where *p<0.05, **p<0.01, ***p<0.001 and ****p<0.0001, n=3. (**B**) CD4+ T cells were activated in vitro with anti-CD3/28 and simultaneously treated with 25-HC or simvastatin. Western blot shows the band for LDLR and GAPDH loading control. (**C**) Resting and activated CD4+ T cells, with lipoproteins or deprived from lipoproteins (lip. depr.). *HMGCR* mRNA was measure with qPCR. Unpaired t test, where *p<0.05, n=3. (**D**) CD25 geometric mean fluorescence intensity (gMFI) measured with flow cytometry on CD4+ T cells activated in vitro for 24 hours. Unpaired t test, where **p<0.01, n=3. (**E**) Representative flow cytometry histograms of the cell trace violet (CTV) dilution. For quantification, we show the proliferation index calculated by the proliferation tool (FlowJo). CD4+ T cells were activated in vitro for four days. Unpaired t test, where **p<0.01, n=3. (**F**) Viability measured on flow cytometry of CD4+ T cells treated with control supernatant of PCSK9 enriched supernatant.

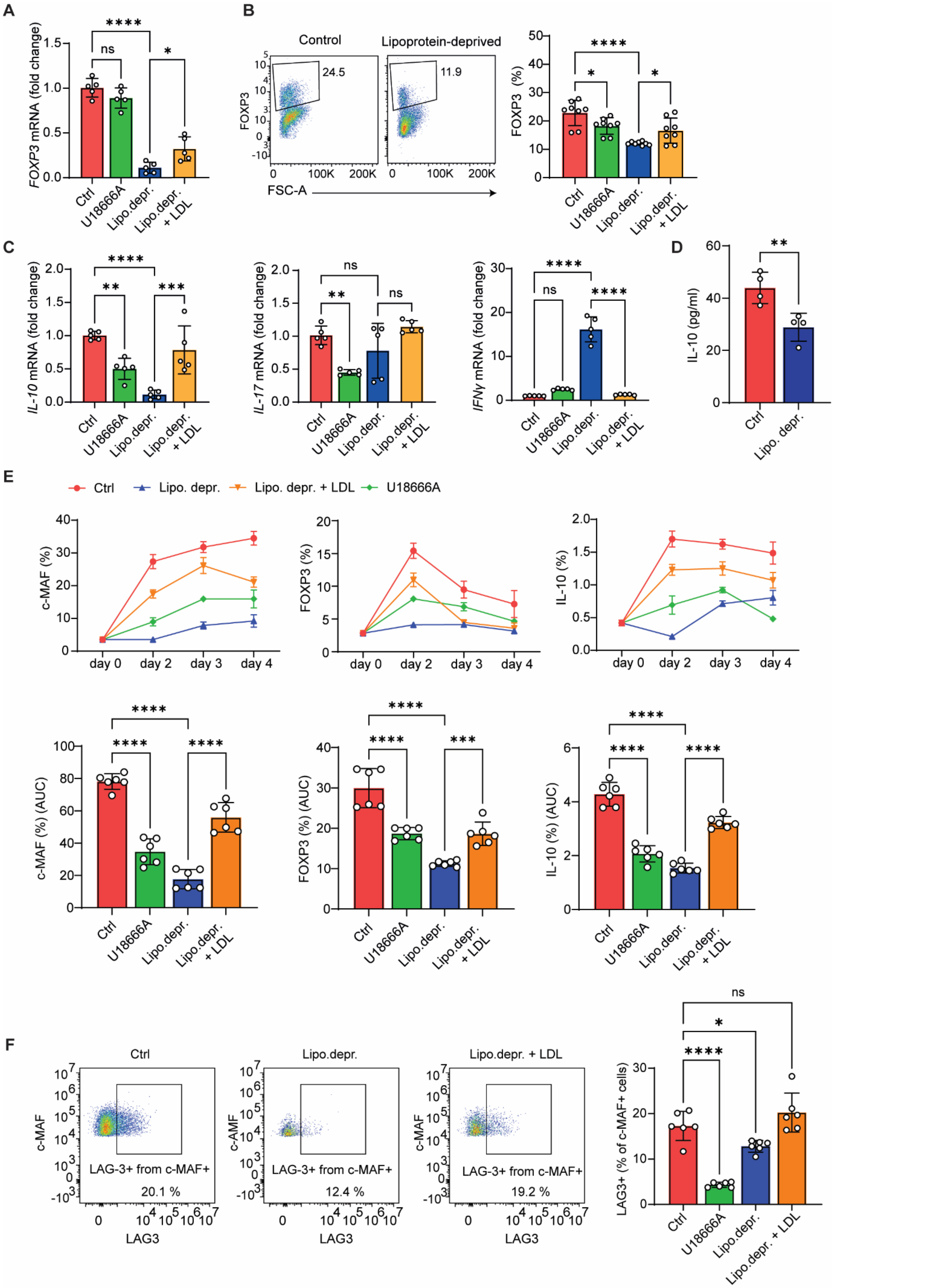

Figure 3. **Lipoproteins are required for CD4+ T cell expression of c-MAF, FOXP3 and IL-10.** (**A**) *FOXP3* mRNA levels measured in CD4+ T cells with qPCR and normalized to RPL13A levels relative to the ctrl condition. One-way ANOVA

with Šídák's multiple comparisons test, where *p<0.05,****p<0.0001, n=5. (**B**) Intracellular protein expression of FOXP3 measured with flow cytometry. One-way ANOVA with Šídák's multiple comparisons test, where *p<0.05, ****p<0.0001, n=8. (**C**) *IFNG, IL17A* and *IL10* mRNA levels measured in CD4+ T cells with qPCR and normalized to RPL13A levels relative to the ctrl condition. One-way ANOVA with Šídák's multiple comparisons test, where **p<0.01, ***p<0.001, ****p<0.0001, n=6. (**D**) IL-10 levels in the supernatants of the monocyte-derived macrophages measured with ELISA. Unpaired t test, where **p<0.01. (**E**) c-MAF, FOXP3 and IL-10 were measured intracellularly using flow cytometry. CD4+ T cells from 3 healthy donors activated with anti-CD3/28 dynabeads. The statistical analysis is done on the area under the curve (AUC). One-way ANOVA with Šídák's multiple comparisons test (***p<0.001 and ****p<0.0001; n=6). (**F**) CD4+ T cells were activated with anti-CD3/28 dynabeads for 48 hours, where indicated also in presence of U18666A, with or without lipoproteins. Intracellular staining was performed for c-MAF and cell surface staining was performed for LAG3 and cells were measured with flow cytometry. Ordinary one-way ANOVA with Dunnett's multiple comparisons test, where *p<0.05, ****p<0.0001 and n=6.

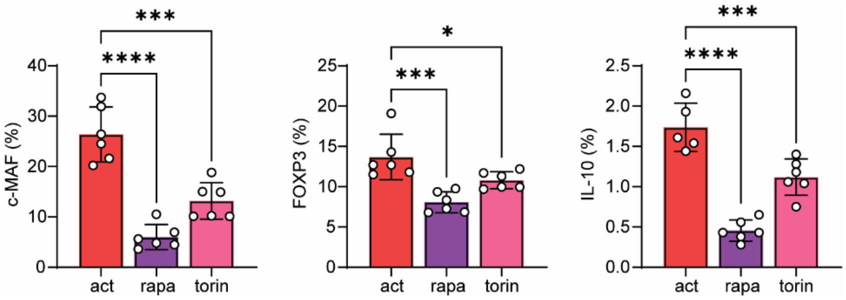

Figure 4. CD4+ T cells were activated with anti-CD3/28 dynabeads for 48 hours, where indicated also in presence of Rapamycin (Rapa) or Torin. Intracellular staining was performed for c-MAF, FOXP3 and IL-10 expression and cells were measured with flow cytometry. Ordinary one-way ANOVA with Dunnett's multiple comparisons test, where *p<0.05, ***p<0.001, ****p<0.0001 and n=6.

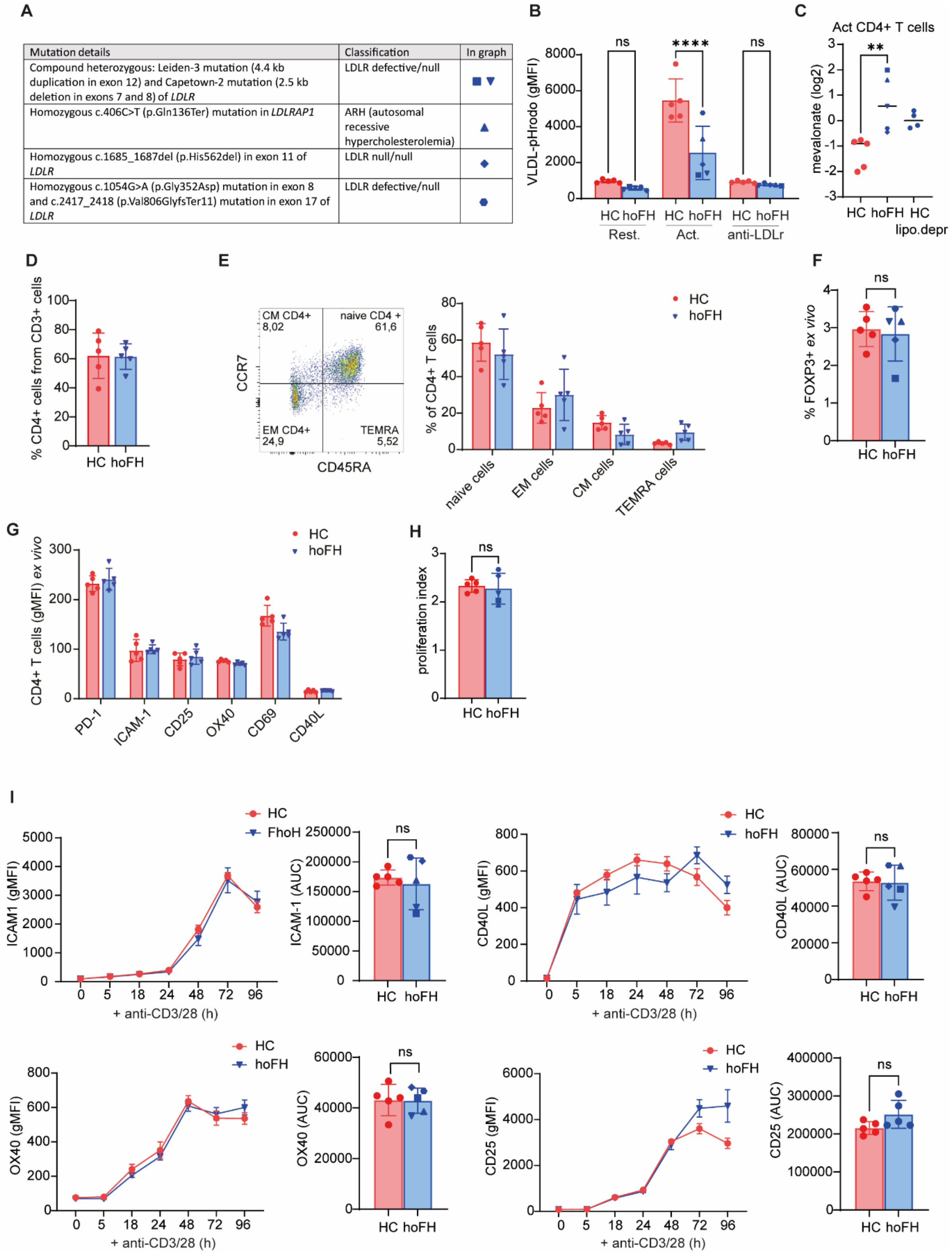

Mutation details	Classification	In graph
Compound heterozygous: Leiden-3 mutation (4.4 kb duplication in exon 12) and Capetown-2 mutation (2.5 kb deletion in exons 7 and 8) of *LDLR*	LDLR defective/null	■ ▼
Homozygous c.406C>T (p.Gln136Ter) mutation in *LDLRAP1*	ARH (autosomal recessive hypercholesterolemia)	▲
Homozygous c.1685_1687del (p.His562del) in exon 11 of *LDLR*	LDLR null/null	◆
Homozygous c.1054G>A (p.Gly352Asp) mutation in exon 8 and c.2417_2418 (p.Val806GlyfsTer11) mutation in exon 17 of *LDLR*	LDLR defective/null	●

Figure 5. CD4+ T cells from homozygous familial hypercholesterolemia (hoFH) patients confirm importance of LDLR signaling for CD4+ T cell function. (**A**) A description of the mutations of patients with hoFH included in our study. (**B**) Uptake of VLDL-pHrodo measured by flow cytometry, where CD4+ T cells were activated for 24 hours, followed by a 2-hour culturing in lipoprotein-deprived medium and 2-hour incubation with the VLDL-pHrodo complex. Where indicated, anti-LDLR (5 µg/ml) was added when cells were cultured in lipoprotein-deprived

medium. One-way ANOVA with Šídák's multiple comparisons test, where ****$p<0.0001$ (n=5 HC and n=5 hoFH). (**C**) Concentration of intracellular mevalonate in CD4+ T cells activated with anti-CD3/28 for 36 hours. Mann–Whitney U test, where **$p<0.01$ (n=5 HC and n=5 hoFH). (**D**) Full PBMCs from healthy controls (HC) and patients with hoFH were stained and measured on flow cytometry for CD3/CD4 expression. (**E**) Ex vivo phenotyping of CD4+ T cells. Cell surface markers were measured with flow cytometry. Central memory (CM) CD4+ T cells are defined as CCR7+ CD45RA-, naïve CD4+ T cells as CCR7+ CD45RA+, effector memory (EM) CD4+ T cells as CCR7- CD45RA- and TEMRA as CCR7- CD45RA+. (**F**) Ex vivo measurement of FOXP3+ cells as percentage of CD4+ T cells. Mann–Whitney U test, where *$p<0.05$ (n=5 HC and n=5 hoFH). (**G**) PD-1, ICAM-1, CD25, OX40, CD69, CD40L were measured on the cell surface of CD4+ T cells with flow cytometry. (**H**) CD4+ T cells from hoFH patients and HC were stained with cell trace violet (CTV) and activated for four days. CTV signal was measured with flow cytometry and for quantification we show the proliferation index calculated by the proliferation tool (FlowJo). Mann–Whitney U test, where *$p<0.05$ (n=5 HC and n=5 hoFH). (**I**) Cell surface expression of ICAM1, CD40L, OX40 and CD25 measured on flow cytometry. The statistical analysis is done on the area under the curve (AUC). Mann–Whitney U test, where *$p<0.05$ (n=5 HC and n=5 hoFH).

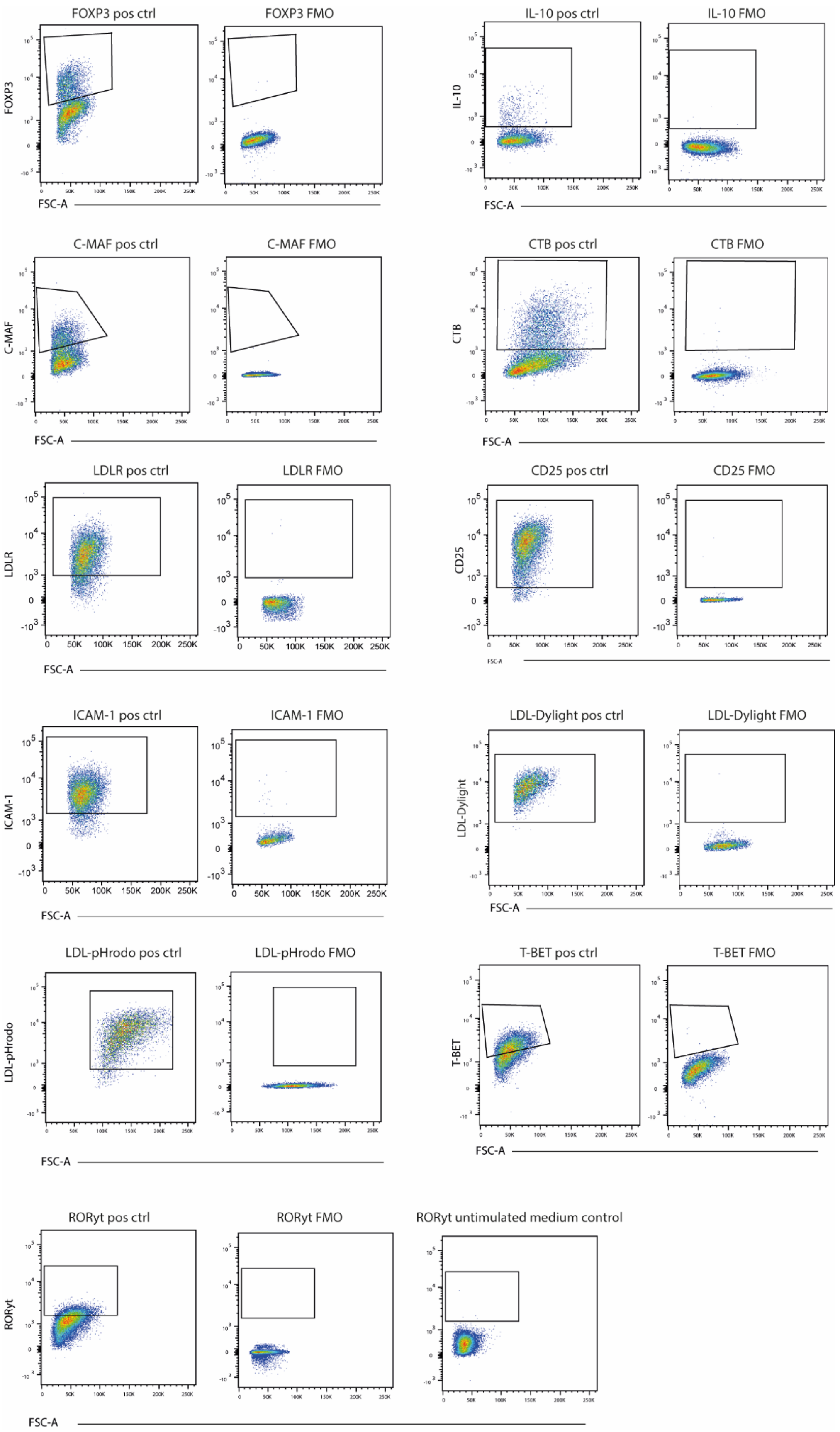

Figure 6. Flow cytometry staining of activated control cells and fluorescence-minus-one (FMO) controls.

Tabe 1. Primer sequences used for SYBR green qPCR

Target gene	Forward primer sequence	Reverse primer sequence
ABCA1	GTTAGGAAACCTGCTGCCCT	ATGCCACACACAGGACGTAG
ABCG1	GGTTCTTCGTCAGCTTCGAC	GTTTCCTGGCATTCAGGTGT
ACACA	CTCTTGGCCTTTTCCCGGTC	GTTATCCCCAAACCCAGGCA
CD36	GGCAGCTGCATCCCATATCT	CCATCTGCAGTATTGTTGTAAGGA
MAF	GGTGTGGGCTTGCTAGTTCT	CCATGAGCCAGACACCCATT
FASN	CTTCAAGGAGCAAGGCGTGA	ACTGGTACAACGAGCGGATG
FOXP3	TCAAGCACTGCCAGGCG	CAGGAGCCCTTGTCGGAT
HMGCR	TTCGGTGGCCTCTAGTGAGA	GATGGGAGGCCACAAAGAGG
IL10	GCCTAACATGCTTCGAGATC	CTCATGGCTTTGTAGATGCC
LDLR	AGCTACCCCTCGAGACAGAT	ACTGTCCGAAGCCTGTTCTG
PLIN2	TGATGGCAGGCGACATCTAC	CTGGCTGCTCTTGTCCATCT
RORC	GAGAAGGACAGGGAGCCAAG	GGATCCCAGACGACTTGTCC
SREBF2	TGAGCCAGGAAGCCCTCTAT	GGGGGTTAAAGGAGAGGCAC
TBX21	GGGAAACTAAAGCTCACAAAC	CCCCAAGGAATTGACAGTTG

Table 2. Antibodies used for flow cytometry staining

Antibody	Clone	Company
Akt-pS473	M89-61	BD
CD127	HCD127	Biolegend
c-MAF	T54-853	BD
FOXP3	PCH101	eBioscience
IL-10	JES3-19F1	Biolegend
LDLR	C7	Bio Connect
Phospho-S6 Ribosomal Protein (Ser235/236)	D57.2.2E	Cell Signaling Technology
Phospho-ZAP70/Syk	n3kobu5	eBioscience
P-p44/42 MAPK (Erk1/2)	197G2	Cell Signaling Technology
RORγt	AFKJS-9	eBioscience
T-bet	4B10	Biolegend
ICAM-1	HCD54	Biolegend
CD25	B1.49.9	Beckman Coulter
