# Supplementary material for: LDL receptor–mediated lipoprotein uptake fuels human CD4+ T cell polarization toward a c-MAF/IL-10– and FOXP3-driven phenotype
Source: JCI Insight. 2026 Jun 8;11(11):e198505. doi: 10.1172/jci.insight.198505 (PMC13313490; doi:10.1172/jci.insight.198505)

Source Western blot images

Fig. 1C

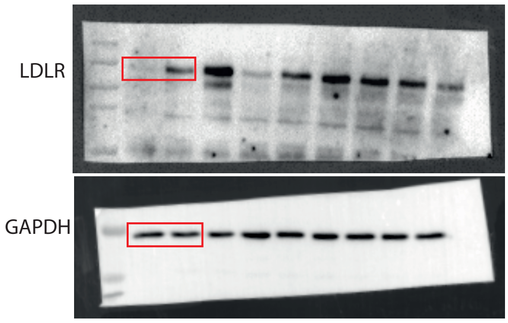

Fig. 2E

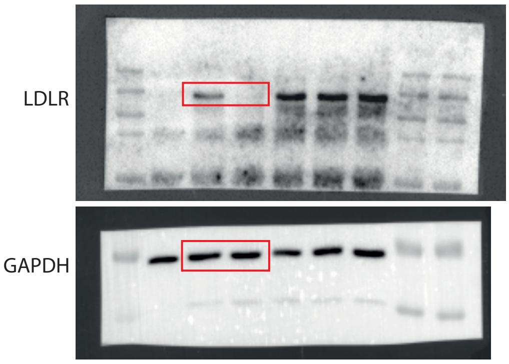

Fig. 5F

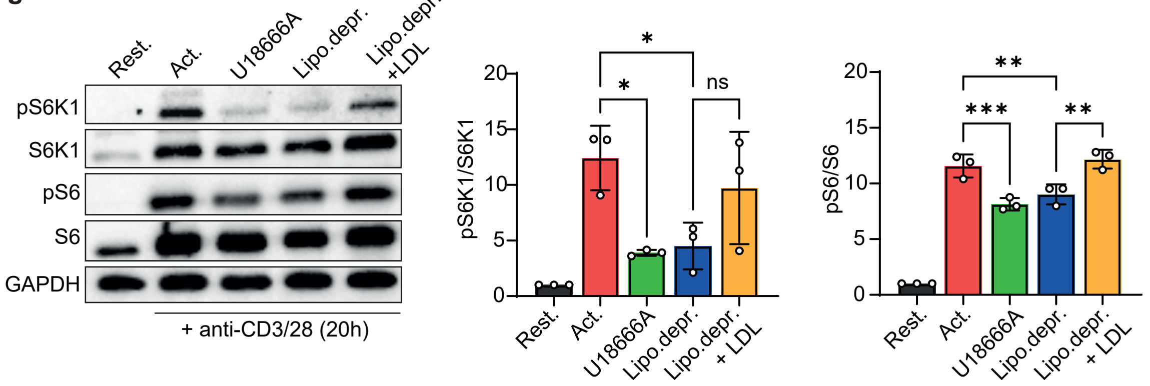

Fig. 5F source for cut Western blot images

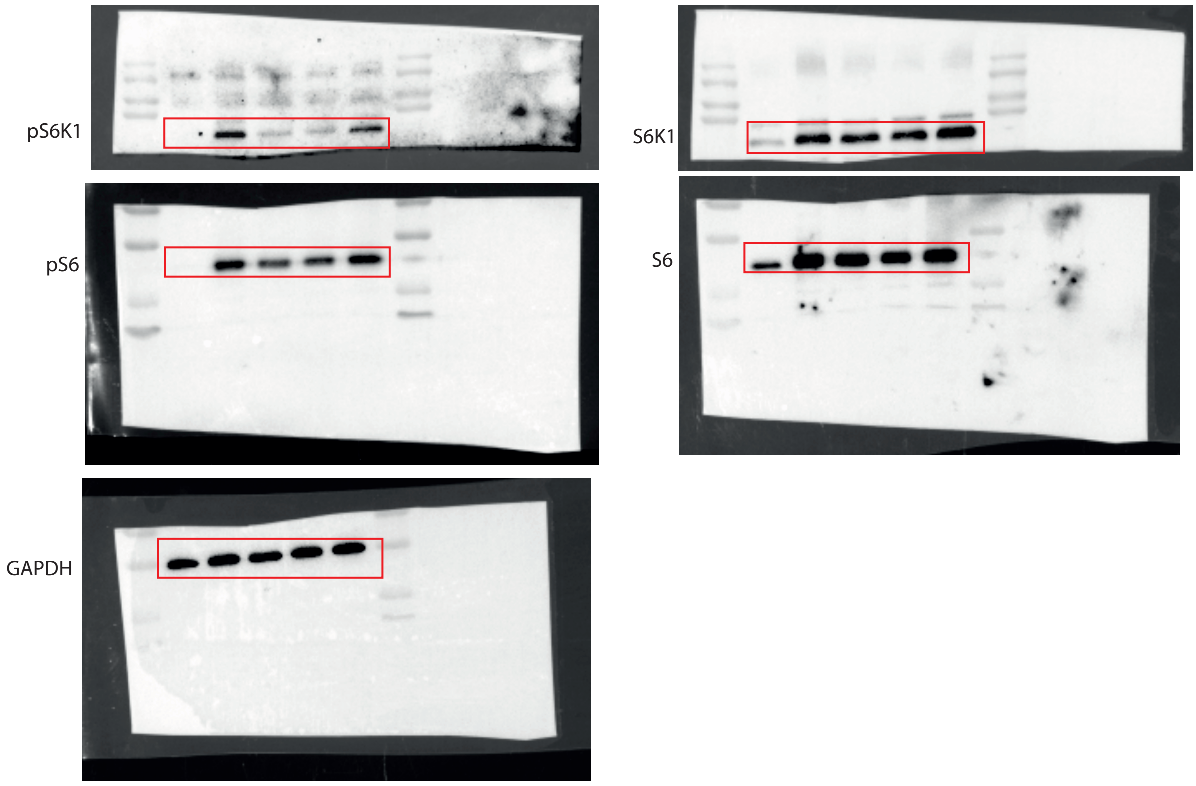

Fig. 5F source for Western blot quantification

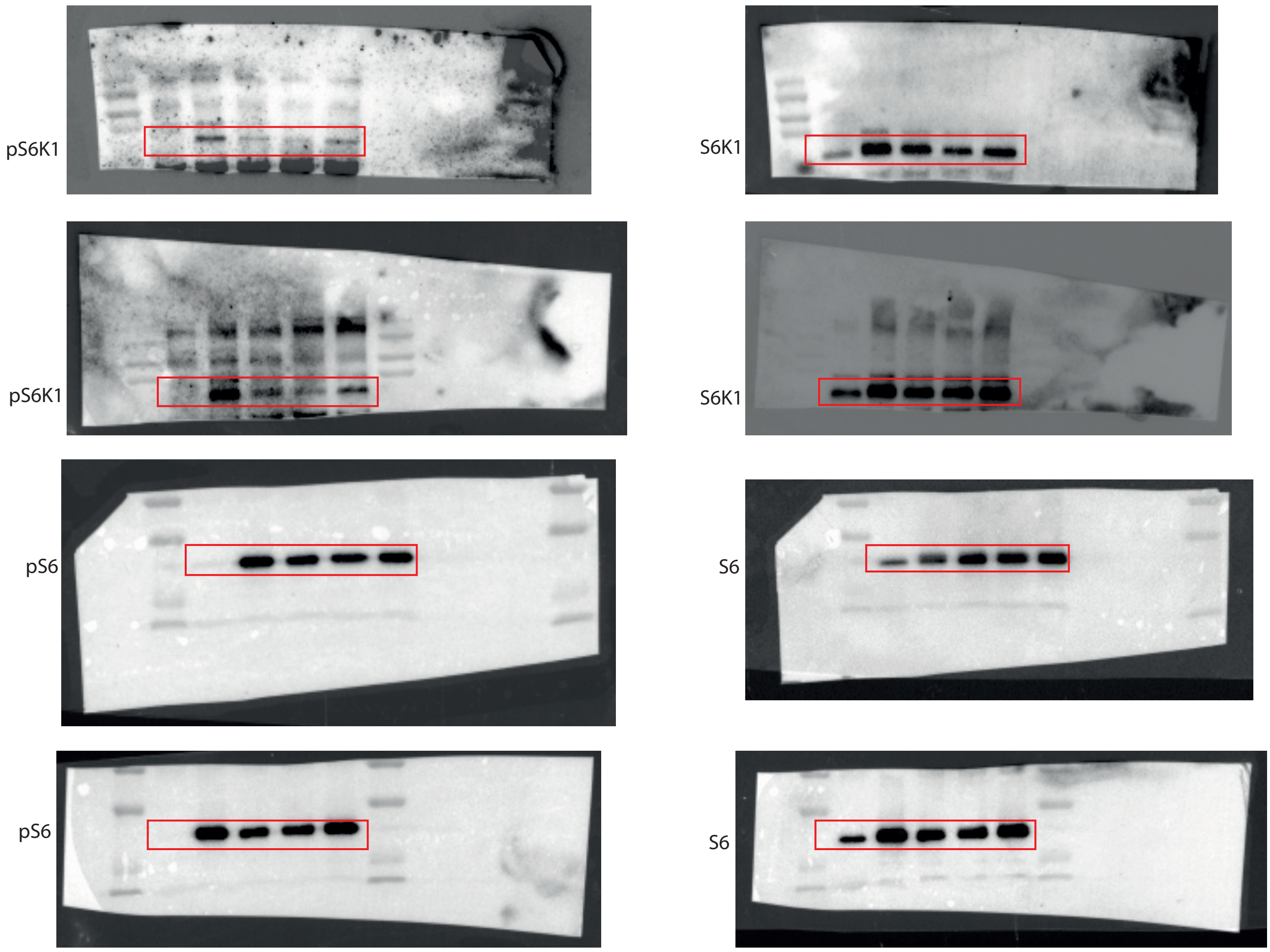

Supplement: Unedited blot and gel images [file jciinsight-11-198505-s116.pdf]
